# The Mitogenome Relationships and Phylogeography of Barn Swallows (*Hirundo rustica*)

**DOI:** 10.1093/molbev/msac113

**Published:** 2022-05-25

**Authors:** Gianluca Lombardo, Nicola Rambaldi Migliore, Giulia Colombo, Marco Rosario Capodiferro, Giulio Formenti, Manuela Caprioli, Elisabetta Moroni, Leonardo Caporali, Hovirag Lancioni, Simona Secomandi, Guido Roberto Gallo, Alessandra Costanzo, Andrea Romano, Maria Garofalo, Cristina Cereda, Valerio Carelli, Lauren Gillespie, Yang Liu, Yosef Kiat, Alfonso Marzal, Cosme López-Calderón, Javier Balbontín, Timothy A. Mousseau, Piotr Matyjasiak, Anders Pape Møller, Ornella Semino, Roberto Ambrosini, Andrea Bonisoli-Alquati, Diego Rubolini, Luca Ferretti, Alessandro Achilli, Luca Gianfranceschi, Anna Olivieri, Antonio Torroni

**Affiliations:** Dipartimento di Biologia e Biotecnologie “Lazzaro Spallanzani”, Università di Pavia, 27100 Pavia, Italy; Dipartimento di Biologia e Biotecnologie “Lazzaro Spallanzani”, Università di Pavia, 27100 Pavia, Italy; Dipartimento di Biologia e Biotecnologie “Lazzaro Spallanzani”, Università di Pavia, 27100 Pavia, Italy; Dipartimento di Biologia e Biotecnologie “Lazzaro Spallanzani”, Università di Pavia, 27100 Pavia, Italy; Vertebrate Genome Laboratory, The Rockefeller University, New York, NY 10065, USA; Dipartimento di Scienze e Politiche Ambientali, Università degli Studi di Milano, 20133 Milan, Italy; Dipartimento di Biologia e Biotecnologie “Lazzaro Spallanzani”, Università di Pavia, 27100 Pavia, Italy; IRCCS Istituto delle Scienze Neurologiche di Bologna, Programma di Neurogenetica, 40139 Bologna, Italy; Dipartimento di Chimica, Biologia e Biotecnologie, Università di Perugia, 06123 Perugia, Italy; Dipartimento di Bioscienze, Università degli Studi di Milano, 20133 Milan, Italy; Dipartimento di Bioscienze, Università degli Studi di Milano, 20133 Milan, Italy; Dipartimento di Scienze e Politiche Ambientali, Università degli Studi di Milano, 20133 Milan, Italy; Dipartimento di Scienze e Politiche Ambientali, Università degli Studi di Milano, 20133 Milan, Italy; Genomic and Post-Genomic Unit, IRCCS Mondino Foundation, 27100 Pavia, Italy; Genomic and Post-Genomic Unit, IRCCS Mondino Foundation, 27100 Pavia, Italy; IRCCS Istituto delle Scienze Neurologiche di Bologna, Programma di Neurogenetica, 40139 Bologna, Italy; Dipartimento di Scienze Biomediche e Neuromotorie, Università di Bologna, 40139 Bologna, Italy; Department of Academic Education, Central Community College, Columbus, NE 68601, USA; State Key Laboratory of Biocontrol, School of Ecology, Sun Yat-sen University, Guangzhou 510275, China; Israeli Bird Ringing Center (IBRC), Israel Ornithological Center, Tel Aviv, Israel; Department of Zoology, University of Extremadura, 06071 Badajoz, Spain; Department of Wetland Ecology, Estación Biológica de Doñana CSIC, 41092 Seville, Spain; Department of Zoology, University of Seville, 41012 Seville, Spain; Department of Biological Sciences, University of South Carolina, Columbia, SC 29208, USA; Institute of Biological Sciences, Cardinal Stefan Wyszyński University in Warsaw, 01-938 Warsaw, Poland; Ecologie Systématique Evolution, Université Paris-Sud, CNRS, AgroParisTech, Université Paris-Saclay, 91405, Orsay Cedex, France; Dipartimento di Biologia e Biotecnologie “Lazzaro Spallanzani”, Università di Pavia, 27100 Pavia, Italy; Dipartimento di Scienze e Politiche Ambientali, Università degli Studi di Milano, 20133 Milan, Italy; Department of Biological Sciences, California State Polytechnic University - Pomona, Pomona, CA 91767, USA; Dipartimento di Scienze e Politiche Ambientali, Università degli Studi di Milano, 20133 Milan, Italy; Dipartimento di Biologia e Biotecnologie “Lazzaro Spallanzani”, Università di Pavia, 27100 Pavia, Italy; Dipartimento di Biologia e Biotecnologie “Lazzaro Spallanzani”, Università di Pavia, 27100 Pavia, Italy; Dipartimento di Bioscienze, Università degli Studi di Milano, 20133 Milan, Italy; Dipartimento di Biologia e Biotecnologie “Lazzaro Spallanzani”, Università di Pavia, 27100 Pavia, Italy; Dipartimento di Biologia e Biotecnologie “Lazzaro Spallanzani”, Università di Pavia, 27100 Pavia, Italy

**Keywords:** barn swallow phylogeny, *Hirundo rustica* subspecies, mitogenome, haplogroups

## Abstract

The barn swallow (*Hirundo rustica*) poses a number of fascinating scientific questions, including the taxonomic status of postulated subspecies. Here, we obtained and assessed the sequence variation of 411 complete mitogenomes, mainly from the European *H. r. rustica*, but other subspecies as well. In almost every case, we observed subspecies-specific haplogroups, which we employed together with estimated radiation times to postulate a model for the geographical and temporal worldwide spread of the species. The female barn swallow carrying the *Hirundo rustica* ancestral mitogenome left Africa (or its vicinity) around 280 thousand years ago (kya), and her descendants expanded first into Eurasia and then, at least 51 kya, into the Americas, from where a relatively recent (<20 kya) back migration to Asia took place. The exception to the haplogroup subspecies specificity is represented by the sedentary Levantine *H. r. transitiva* that extensively shares haplogroup A with the migratory European *H. r. rustica* and, to a lesser extent, haplogroup B with the Egyptian *H. r. savignii*. Our data indicate that *rustica* and *transitiva* most likely derive from a sedentary Levantine population source that split at the end of the Younger Dryas (YD) (11.7 kya). Since then, however, *transitiva* received genetic inputs from and admixed with both the closely related *rustica* and the adjacent *savignii*. Demographic analyses confirm this species’ strong link with climate fluctuations and human activities making it an excellent indicator for monitoring and assessing the impact of current global changes on wildlife.

## Introduction

The barn swallow (*Hirundo rustica*) is one of the most widely distributed bird species (Turner and Rose 2010), possibly due to the switch from natural nesting sites, especially caves, to nesting in human-made structures (Zink et al. 2006). This commensal and iconic species for numerous human groups and cultures is portrayed in art, myths, legends, and poetry for millennia (Green 1988) and comprises at least six subspecies, all with breeding ranges in the Holarctic (but see Areta et al. 2021). The subspecies differ in several morphometric characteristics, such as body size, length of outer tail streamers, ventral coloration, and extent of the dark breast band (Turner 2006). The subspecies include *H. r. rustica* (Europe, North Africa and Western Asia), *H. r. savignii* (Egypt), *H. r. transitiva* (Israel, Lebanon, Jordan and Syria), *H. r. tytleri* (southern-central Siberia, Mongolia), *H. r. gutturalis* (central-eastern China, Japan), and *H. r. erythrogaster* (North America). Additional subspecies such as *H. r. saturata* and *H. r. mandshurica* have been postulated in north-eastern Asia, but their distinct subspecies status relative to the other Asian subspecies is debated (Cheng 1987; Brown and Brown, 1999; Dickinson and Dekker, 2001; Dickinson et al. 2002; Turner 2006; Liu et al. 2020). While the *Hirundo rustica* species complex is not endangered, local populations or even subspecies show declines due to specific threats, mostly related to agricultural intensification (Ambrosini et al. 2012; Møller 2019). Most subspecies are migratory, and their wintering grounds cover much of the southern hemisphere as far south as central Argentina, the Cape province of South Africa, and northern Australia (Turner 2006; Hobson et al. 2015; Liechti et al. 2015; Winkler et al. 2017). Adult swallows are highly philopatric (Møller 1994), whereas natal dispersal is relatively large, with some individuals, especially females, dispersing up to several hundreds of kilometers from their natal site (Turner 2006; Balbontín et al. 2009; Scandolara et al. 2014). However, *H. r. savignii* and *H. r. transitiva* are sedentary throughout the year (Shirihai et al. 1996; Turner 2006; Turner and Rose 2010), or make short-distance movements during the non-breeding period (Kiat, unpublished data).

The earliest study of barn swallow nuclear DNA variation (*MUSK* gene) did not detect a genetic structure within the species, suggesting a rather recent subspecies differentiation (Zink et al. 2006). More recent and extensive surveys of microsatellite and double digest Restriction-site Associated DNA (ddRAD) sequence data in *H. r. rustica* revealed a lack of population structure among breeding populations from Sweden, Germany, and Switzerland with no evidence of genomic selection between phenotypic migratory types (Santure et al. 2010; von Rönn et al. 2016). In contrast, genotyping of over 9,000 Single-Nucleotide Polymorphisms (SNPs) in 350 barn swallows from four subspecies revealed genome-wide clustering that generally corresponds with the subspecies, a certain level of differentiation of the UK population (*H. r. rustica*) from eastern European and Turkish populations of the same subspecies and genomic covariance of the latter *H. r. rustica* populations with non-migratory *H. r. transitiva* specimens from Israel (Safran et al. 2016). With a similar approach, molecular evidence of hybridization between subspecies was also obtained (Scordato et al. 2017; 2020).

In the last few years, whole genome sequencing data have been reported for a few subspecies (*H. r. erythrogaster*, *H. r. savignii*) (Safran et al. 2016; Smith et al. 2018), including the first reference genome draft (*H. r. rustica*) (Formenti et al. 2019). Recently, in the framework of the Vertebrate Genomes Project, an effort to generate complete and accurate genome assemblies for all vertebrate species, a new reference genome for *H. r. rustica* as well as the first pangenome for the species was released. This allowed the assessment of the extent of conservation and acceleration in the barn swallow genome and the identification of a catalogue of genetic markers and candidate genomic regions under selection (G. Formenti, data not shown).

So far, however, most genetic studies concerning the relationships between barn swallow subspecies have focused on the maternally transmitted and fast-evolving mitochondrial DNA (mtDNA), particularly on the sequence variation of single mitochondrial genes, such as *ND2* and *CYB* (Sheldon et al. 2005; Zink et al. 2006; Dor et al. 2010, 2012; Malaitad et al. 2016). They confirmed that the barn swallow species complex is monophyletic, and revealed that the different subspecies cluster into two major phylogenetic branches, which might have diverged approximately 100 thousand years ago (kya) and geographically correspond to Europe-Middle East and Asia-America (Zink et al. 2006; Dor et al. 2010), thus substantially predating human agriculture and the new nesting opportunities provided by human settlements. Moreover, the close relationship between one of the Asian subspecies (*H. r. tytleri*) and the American one (*H. r. erythrogaster*) has raised the possibility of a secondary dispersal event, possibly about 27 kya, from North America back into Asia (Zink et al. 2006). Finally, the potential lack of differentiation between the migratory *H. r. rustica* and the sedentary *H. r. transitiva* was also observed with the fast-evolving mtDNA (Dor et al. 2010), suggesting intermingling between the two subspecies.

Despite the valuable genetic insights provided by these studies, the assessment of only a rather short segment of the barn swallow mtDNA limits their phylogenetic resolution and the understanding of this species’ origin and spread. A finer phylogenetic resolution can be achieved by sequencing the entire mitogenome, an approach that has been employed for humans and many other animal species (Achilli et al. 2008, 2012; Behar et al. 2012; Miao et al. 2013; Morin et al. 2015; Battaglia et al. 2016; Barth et al. 2017; Peng et al. 2018; Cole et al. 2019; de Manuel et al. 2020; Niedziałkowska et al. 2021) and recently pursued also in *H. rustica* (Carter et al. 2020). Here, we exploited next generation sequencing (NGS) to obtain 411 complete mitogenomes, mainly from the European *H. r. rustica*, but also from other subspecies. Phylogenetic and Bayesian analyses allowed us to 1) obtain a high-resolution mitogenome phylogeny of the species, 2) better define the matrilineal relationships and links between subspecies and their divergence times, and 3) assess demography through time.

## Results and Discussion

### Organization of the Barn Swallow Mitogenome

Our first complete mitogenome was obtained from a *H. r. rustica* specimen (no. 20) from Italy (supplementary table S1, Supplementary Material online). This mitogenome (MZ905359), employed as *H. r. rustica* Reference Sequence (HrrRS), was Sanger sequenced together with four additional *H. r. rustica* mitogenomes from Italy (nos. 1, 35, 136, and 302). The mitogenome is 18.143 bps in length and harbors 37 genes: 13 protein-coding, 22 tRNA, and two rRNA genes, as well as two non-coding regions, CR1 and CR2, following the GO-II gene order (Mackiewicz et al. 2019; Urantówka et al. 2020) (supplementary fig. S1 and supplementary table S2, Supplementary Material online).

**Fig. 1.**
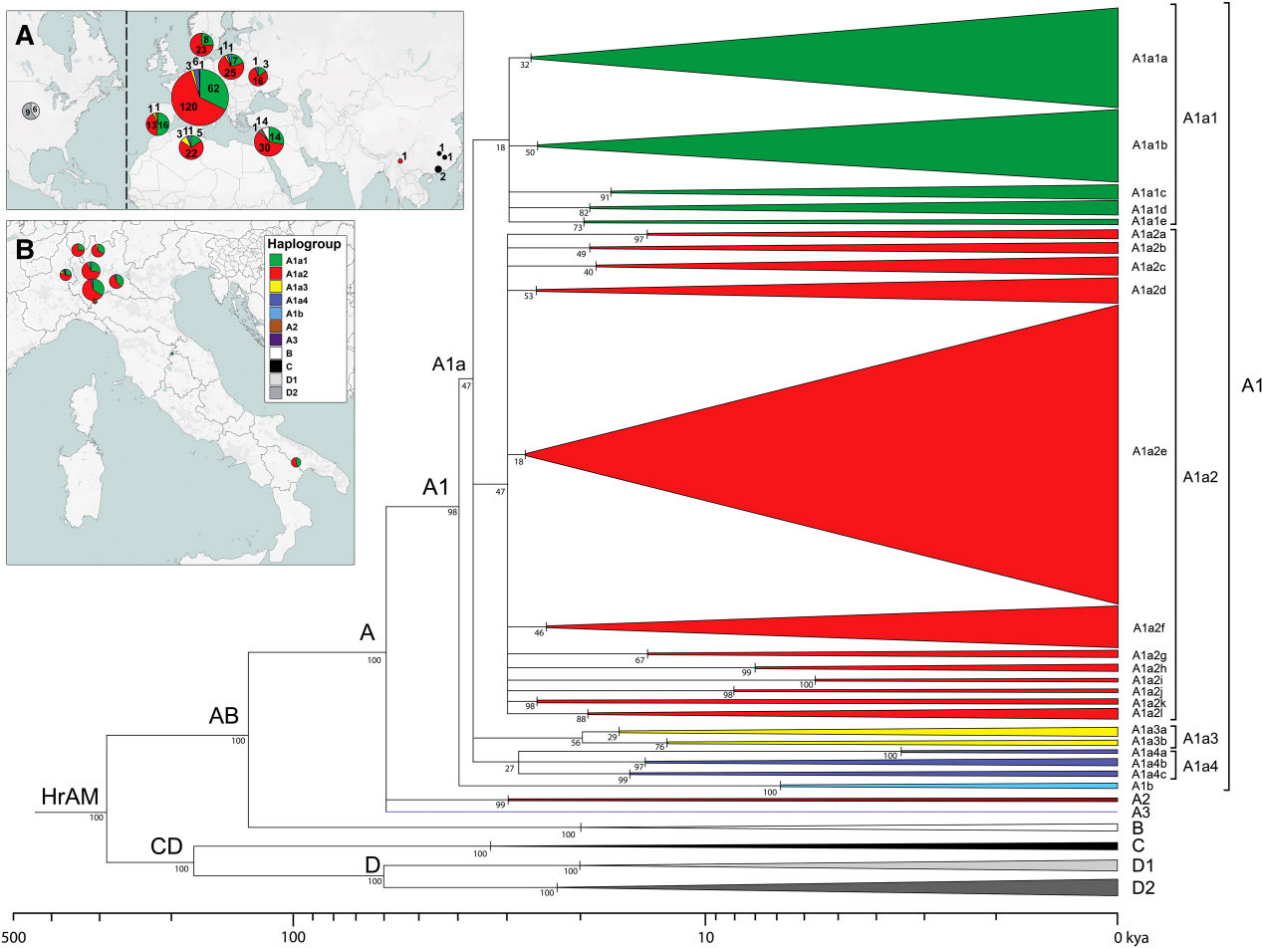
Schematic MP phylogeny of *Hirundo rustica* mitogenomes. This tree was built using the entire mitogenome coding-region (15,601 bps; nps 1–14,859, nps 16,068–16740, nps 18,075–18,143) of 411 barn swallows. It was rooted using *H. angolensis* (NC_050287) and *H. aethiopica* (NC_050293) reference mitogenomes (not displayed). Haplogroups are represented as triangles whose bases are proportional to the number of mitogenomes. HrAM refers to the *Hirundo rustica* Ancestral Mitogenome. Different colors were assigned only to major branches. Bootstrap values (1000 iterations) are shown. The timeline (log_10_) at the bottom refers to the Bayesian coalescence times of supplementary table S3, Supplementary Material online. The insets (*A*) and (*B*) illustrate the frequencies of the major haplogroups in the different sampling locations. Inset (*B*) details frequencies in Italy and Switzerland.

NGS technology was employed to sequence additional 405 entire barn swallow mitogenomes and another was extrapolated from Formenti et al. (2019). These mitogenomes were from four putative subspecies (336 *H. r. rustica*, 50 *H. r. transitiva*, 5 *H. r. gutturalis*, and 15 *H. r. erythrogaster*); for *H. r. rustica*, they were from numerous sampling locations (fig. 1; supplementary fig. S2 and supplementary table S1, Supplementary Material online). A total of 387 distinct haplotypes were detected, with 1,385 variable sites in the coding region (15,601 bps; nps 1–14,859, nps 16,068–16,740, nps 18,075–18,143). On average, 32.8 ± 1.0 nucleotide differences were found between any two coding-region sequences. The average *π* value for the 411 entire mitogenomes is 0.226% (±0.018%) with the highest variability in the two control regions (Supplementary fig. S3, Supplementary Material online). A total of 1102 synonymous and 156 non-synonymous mutations were identified in the 13 protein-coding genes (PCGs) (supplementary fig. S4, Supplementary Material online). As expected, all loci harbor more synonymous than non-synonymous mutations indicating the action of purifying selection (Stewart et al. 2008).

### The Phylogeography of Barn Swallow Mitogenomes and Haplogroup Ages

Phylogenetic analyses reveal that all 411 *Hirundo rustica* mitogenomes cluster into four main branches that we named haplogroups A, B, C, and D (fig. 1; supplementary fig. S5, Supplementary Material online). These mitogenomes derive from a common female ancestor that harbored the *H. rustica* ancestral mitogenome (HrAM). Consistent with previous results, the four haplogroups are included in two primary branches (Zink et al. 2006; Dor et al. 2010) resulting from the first split in the phylogeny. One of the branches includes haplogroups A and B, and the other encompasses haplogroups C and D. We thus named them AB and CD, respectively. As previously noted (Dor et al. 2010), this division is supported by a plumage trait, the dark breast band, which is broad and complete in the subspecies clustering within the AB branch (*H. r. rustica*, *H. r. transitiva, H. r. savignii*), and narrow or incomplete in those with CD mitogenomes (*H. r. gutturalis, H. r. erythrogaster*, *H. r. tytleri*).

For all nodes in the phylogeny and the derived haplogroups and sub-haplogroups, we obtained age estimates both with maximum likelihood (ML) and Bayesian approaches. The estimates obtained with the two methods are very similar (supplementary table S3, Supplementary Material online). Thus, for brevity we report here only the Bayesian ages.

According to our data, the female barn swallow carrying the HrAM lived 276.9 ± 24.3 kya, an almost three-fold age increase relative to earlier estimates (Zink et al. 2006; Dor et al. 2010). A result of this type was not unexpected. Indeed, by improving the molecular and phylogenetic resolution of mtDNA to the level of entire (or almost entire) mitogenomes, important age changes for the most recent common female ancestor were reported in different species (Achilli et al. 2012), including humans (Torroni et al. 2006; Behar et al. 2012).

**Fig. 2.**
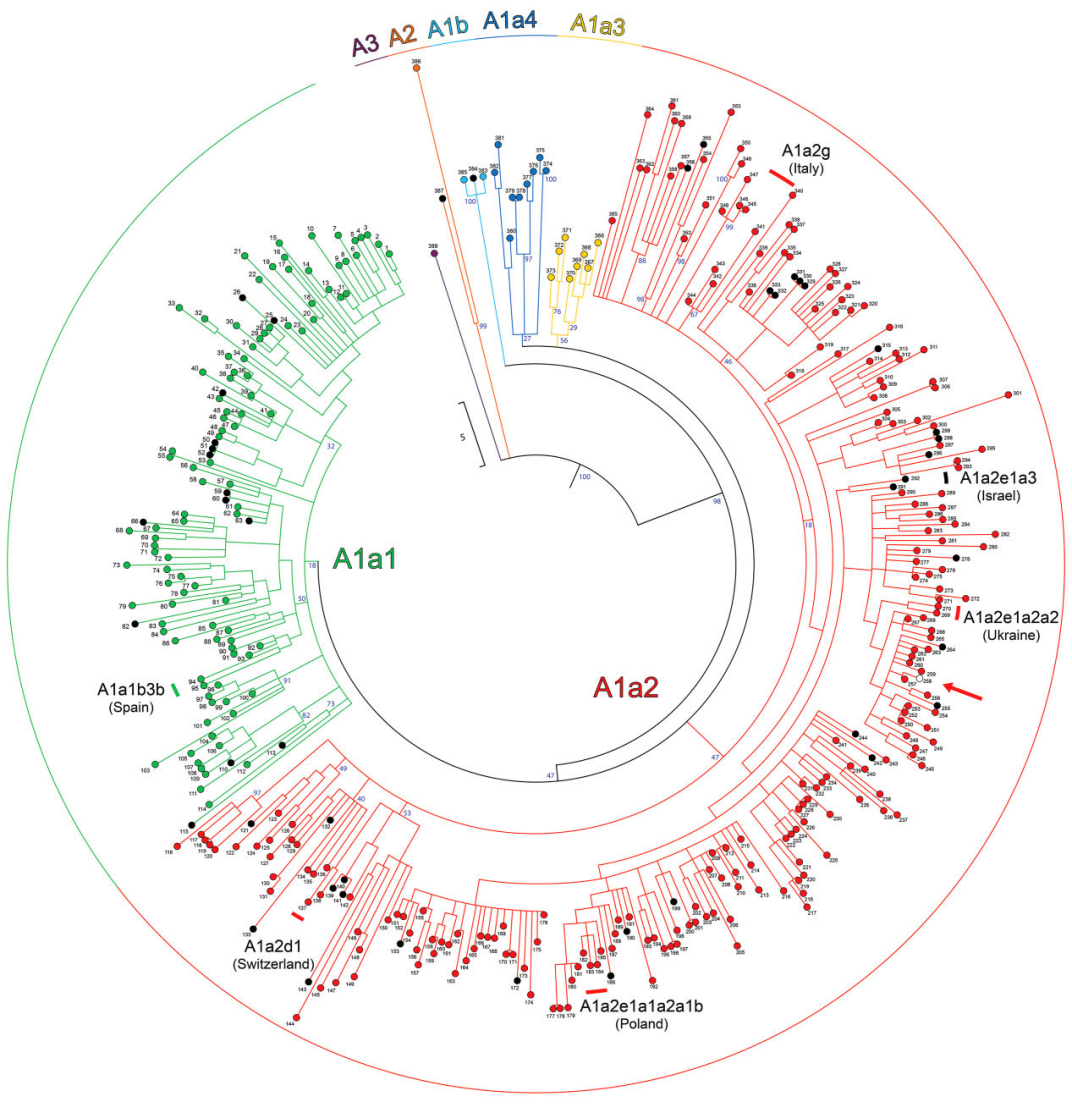
Schematic MP phylogeny of haplogroup A mitogenomes. This tree was built using the coding regions of 388 mitogenomes belonging to haplogroup A (fig. 1) and was rooted with the available *H. angolensis* (NC_050287) and *H. aethiopica* (NC_050293) reference mitogenomes (not displayed). Mitogenomes marked in black are from *H. r. transitiva* specimens sampled in Israel, the one in white (see also red arrow) is from a *H. r. gutturalis* sample (no. 258) from China, while all others are from *H. r. rustica*. Main haplogroup affiliations are shown, with branches colored according to fig. 1. Branch lengths are proportional to the number of nucleotide substitutions. Bootstrap values (1000 iterations) are shown only for the deepest nodes. Six country-specific sub-haplogroups are also shown. They are the oldest found in the reported country. Additional details about samples and mitogenomes are provided in supplementary table S1, Supplementary Material online.

Of the four main haplogroups, haplogroup A is by far the best represented (*n* = 388) in our sample (figs. 1 and 2). It began to radiate 57.1 ± 6.4 kya and comprises all mitogenomes from Europe and Algeria (*H. r. rustica*) as well as 46 of the 50 *H. r. transitiva* mitogenomes from Israel and one from *H. r. gutturalis* (no. 258) sampled in China (Nujiang Prefecture, Yunnan Province). Three sub-haplogroups originated from its initial split, the largely predominant A1 and the rare A2 and A3, with the former harboring two very common sub-branches detected in all European locations as well as in Algeria (fig. 1), with mitogenomes from each location generally scattered and intermingled with those from the other European locations. Furthermore, we observed that the 46 mitogenomes from *H. r. transitiva* belonging to A (black dots in fig. 2) are also scattered among the *H. r. rustica* mitogenomes.

These observations tend to confirm the rather poor genetic differentiation of *H. r. rustica* populations at a high level of molecular and phylogenetic resolution and of *H. r. transitiva* too, at least for the predominant haplogroup A component. Our *H. r. transitiva* sample from Israel would be essentially indistinguishable from the European *H. r. rustica* populations, if not for the detection of four haplogroup B (8.0%) mitogenomes (fig. 1; supplementary table S1 and supplementary fig. S5, Supplementary Material online). A diffuse and broad overlap of the mtDNA variation between *H. r. rustica* and *H. r. transitiva* is also confirmed by the haplogroup A diversity values in the two subspecies, which are identical (0.13%) (supplementary table S4, Supplementary Material online). Three possible explanations can be envisioned for the extensive mtDNA overlap between *rustica* and *transitiva*. First, the two adjacent subspecies derive from the same ancestral source in which A was the only (or predominant) haplogroup and was already differentiated into sub-haplogroups at the time of the initial *rustica*-*transitiva* split. Alternatively, *rustica* and *transitiva* maternal lineages underwent gene flow, possibly continuously over time. Finally, *rustica* and *transitiva* derive from the same ancestral population, but also admix; a process that is still going on, despite the (growing) differences in migratory behavior, moult strategy (Kiat et al. 2019) and morphology, when migrant *rustica* individuals pass through the *transitiva* breeding areas at the main time of their breeding season.

Nevertheless, because of the abundance of haplogroup A mitogenomes in our collection, we also detected a certain amount of genetic differentiation among populations. Indeed, a number of subclades harbor rather localized geographic distributions and appear to be population-specific. These subclades are not uncommon and sometimes they are relatively ancient: four were found in Spain (2–3 haplotypes each) with the oldest (A1a1b3b) dating ∼11.4 ky; 20 in Italy (2–5 haplotypes each) with the oldest (A1a2g) dating ∼11.6 ky; one (A1a2d1, 2 haplotypes) in Switzerland dating ∼8.0 ky; two (2 haplotypes each) in Ukraine with the oldest subclade (A1a2e1a2a2) dating ∼7.6 ky; and one (3 haplotypes) in Poland (A1a2e1a1a2a1b) dating ∼6.0 ky. This feature is not exclusive to *H. r. rustica*, but it characterizes also *H. r. transitiva*: two subclades (2–3 haplotypes) with the oldest (A1a2e1a3) dating ∼11.6 ky (supplementary table S3, Supplementary Material online).

With a lower degree of specificity, some geographic clustering characterizes also a few more common and sometimes older branches. For example, sub-haplogroups A1a1a1a (∼19.5 ky), A1a2e1a1a5 (∼11.5 ky), and A1a2f1b (∼11.1 ky) are over-represented in the Danish population (*χ*^2^  _[24]_ = 10.276, 29.752 and 14.970; *P* = 0.0028, 0.0001 and 0.0032, respectively) compared to other European locations (supplementary fig. S6, Supplementary Material online).

At the other extreme, we also observed a couple of instances in which specimens sampled at very distant locations harbored the same haplotype (no. 177 from Denmark and no. 178 from Italy; no. 200 from Poland and no. 201 from Italy). They suggest that long-distance dispersal between populations occurs, in agreement with observations concerning the behavioral flexibility and adaptability of the species (Mead 2002; Turner 2006; Romano et al. 2017; Teglhøj 2020).

While limited by the relatively small size of our population samples and restricted to haplogroup A mitogenomes, the complete or partial clustering of some sub-haplogroups of A would fit with the generally reported short-distance dispersal of offspring from natal to breeding sites, although this feature is less extreme in females compared to males (Balbontín et al. 2009; Scandolara et al. 2014), thus less detectable in terms of mtDNA. On the other hand, the general overall sharing of the haplogroup A branches among *H. r. rustica* populations and between *H. r. rustica* and *H. r. transitiva* can be at least in part explained when considering that even short-distance dispersal can lead to extensive and long-distance gene flow over the course of generations. Moreover, if the instances of long-distance dispersal from natal to breeding sites are confirmed, even at a low percentage, they would further speed up the loss of genetic structure in European barn swallow populations.

As for the remaining three major haplogroups, B, C, and D (fig. 1; supplementary fig. S5, Supplementary Material online), the former encompasses only the four *H. r. transitiva* mitogenomes already mentioned above and is dated at 18.9 ± 3.9 kya. It shares an ancestral node (AB; 115.6 ± 13.3 kya) with the sister haplogroup A, which is approximately 40 ky younger than the CD node (156.4 ± 18.0 kya) from which C and D derive.

Haplogroup C includes only *H. r. gutturalis* samples, four of the five sampled in China and is dated at 31.1 ± 5.7 kya, while the fifth is a member of haplogroup A. The detection of haplogroup A in the *gutturalis* individual might indicate past or present admixture with *rustica*, especially when considering that it was collected in the westernmost (Nujiang Prefecture) of the sampling locations in China, the closest to the breeding range of *H. r. rustica*.

Finally, haplogroup D, dated at 51.1 ± 7.9 kya, characterizes all 15 *H. r. erythrogaster* specimens from North America (USA, Nebraska), in either one or the other of its sub-haplogroups (D1 and D2). Haplogroup D age provides a minimum time for the spread of *H. rustica* from Asia to the Americas and indicates that North America was most likely the nesting ground of the ancestors of *H. r. erythrogaster* since at least 51 ky ago.

### Subspecies Specificity of the Major Haplogroups

**Fig. 3.**
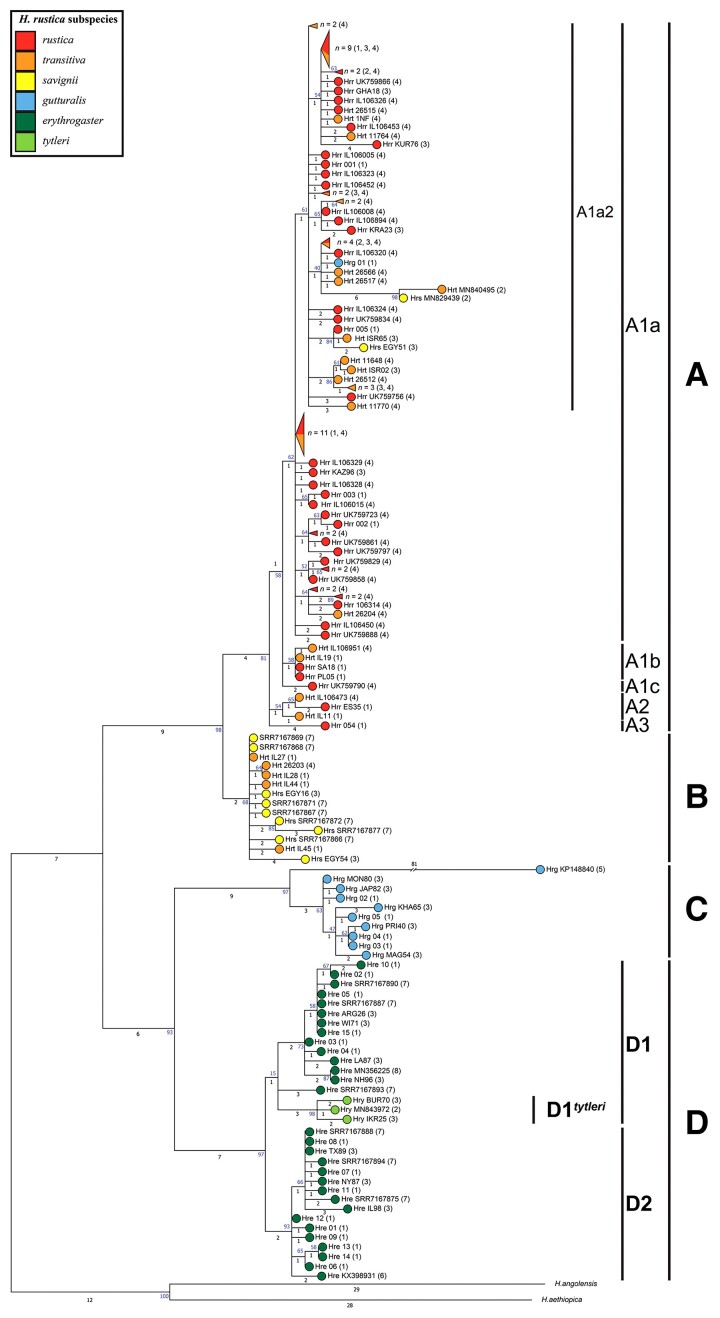
MP phylogeny of *Hirundo rustica ND2* and *CYB* gene sequences. This tree includes 155 barn swallows from different subspecies for which both *ND2* and *CYB* gene sequences were available. A total of 119 are from the literature and the remaining were selected from our mitogenome dataset as follows: the first five mitogenomes that we obtained from *H. r. rustica* (nos. 1, 20, 35, 136, 302) and the one from Formenti et al. (2019) (no. 151), all mitogenomes from the uncommon sub-haplogroups of A (A1b, A2, A3; nos. 383–388) and all mitogenomes belonging to haplogroups B, C and D (nos. 389–411) (supplementary table S1, Supplementary Material online). Sequences encompass 2,075 bps, 1,017 bps of *ND2* (nps 3,980–4,996) and 1,058 bps of *CYB* (nps 13,696–14,753). The tree was rooted using the *H. aethiopica* and *H. angolensis* reference mitogenomes. Main haplogroup and sub-haplogroup affiliations are shown. Colors identify the different subspecies. The numbers on the branches indicate the number of distinguishing mutations while the numbers in parentheses refer to the following publication sources: 1) this study; 2) Carter et al. (2020); 3) Dor et al. (2010); 4) Dor et al. (2012); 5) Liu et al. (2015), direct submission; 6) Keepers et al. (2016), direct submission; 7) Smith et al. (2018); 8) Feng et al. (2020). Sequences not covering the aforementioned *ND2* and *CYB* gene ranges were not included, as well as sequences that harbored gaps at informative nucleotides. The two mtDNAs forming the rather long sub-branch (6 mutations) within A1a2, one from *H. r. savignii*, and one from *H. r. transitiva* (Carter et al. 2020), most likely contain erroneous mutations as their mitogenome sequences harbored NUMTs (see Materials and Methods). A similar problem characterizes the *H. r. gutturalis* sequence KP148840 (Liu et al. 2015, direct submission) with its 81 mutations branch.

To gain a broader view of the haplogroup distribution in the different subspecies, including some not included in our study, we compared the combined *ND2* and *CYB* gene variation of our mitogenomes with that reported in 119 barn swallow mtDNAs available from previous studies (Dor et al. 2010, 2012; Liu et al. 2015, direct submission; Keepers et al. 2016, direct submission; Smith et al. 2018; Feng et al. 2020; Carter et al. 2020) (fig. 3).

The phylogeny of fig. 3 confirms that haplogroup A is typical of both *H. r. rustica* and *H. r. transitiva*, with *H. r. transitiva* mitogenomes scattered among those of *H. r. rustica* in virtually all sub-haplogroups of A. Moreover, it reveals that the four haplogroup B mitogenomes observed in *H. r. transitiva* form a clade that is defined by the transitions at nps 14,235 and 14,243 in *CYB*. This branch encompasses also an additional *H. r. transitiva* specimen (Dor et al. 2012) and nine of eleven *H. r. savignii* (Dor et al. 2010; Smith et al. 2018). This high frequency of haplogroup B in *H. r. savignii* indicates that haplogroup B is typical of the sedentary Egyptian subspecies. Moreover, the detection of some B mitogenomes in *transitiva* and some A mitogenomes in *savignii* (fig. 3) appears to indicate that gene flow of maternal lineages is not restricted to *transitiva* and *rustica*, but it also occurs between *transitiva* and *savignii*, and possibly also between *rustica* and *savignii.* These and other alternative scenarios cannot be distinguished without nuclear genome data.

As for haplogroup C (*n* = 10), the phylogeny confirms instead its complete subspecies specificity. It includes only *H. r. gutturalis* specimens: the four from China of this study (nos. 393–396), one from Japan, three from Russia, one from Mongolia (Dor et al. 2010), and one of an undefined Asian origin (Liu et al. 2015, direct submission).

A more complex situation concerns haplogroup D. The phylogeny of fig. 3 supports the exclusive affiliation of all *H. r. erythrogaster* specimens (*n* = 30) to haplogroup D: the 15 from Nebraska of this study (nos. 397–411), additional 14 from the USA (Dor et al. 2010; Keepers et al. 2016, direct submission; Smith et al. 2018), and one from Argentina (Dor et al. 2010). As already shown by the phylogeny of entire mitogenomes, they all belong to either sub-haplogroups D1 or D2, whose ages are estimated at 19.7 ± 3.9 kya and 20.6 ± 3.4 kya, respectively (supplementary table S3, Supplementary Material online).

However, *ND2* and *CYB* sequences are available also for three *H. r. tytleri* specimens (Dor et al. 2010; Carter et al. 2020), an Asian subspecies that was not included in our survey of entire mitogenomes. The three *H. r. tytleri* partial mtDNA sequences appear to form a private sub-haplogroup within D1, which we named D1*^tytleri^* (fig. 3). It is a sister branch to the D1 branches of *H. r. erythrogaster*, thus supporting the previously proposed close relationship between *H. r. tytleri* and *H. r. erythrogaster* as well as an American origin of the ancestral mitogenomes of *H. r. tytleri* (Zink et al. 2006; Dor et al. 2010, 2012). Moreover, taking into consideration that D1 arose approximately 20 kya, we have now a maximum age boundary for the back migration from North America: the ancestors of *H. r. tytleri* did not move to Asia earlier than 20,000 years ago. As for the minimum boundary for this event, it will be defined only by sequencing *H. r. tytleri* mitogenomes.

### The Demography of Barn Swallows Over Time

**Fig. 4.**
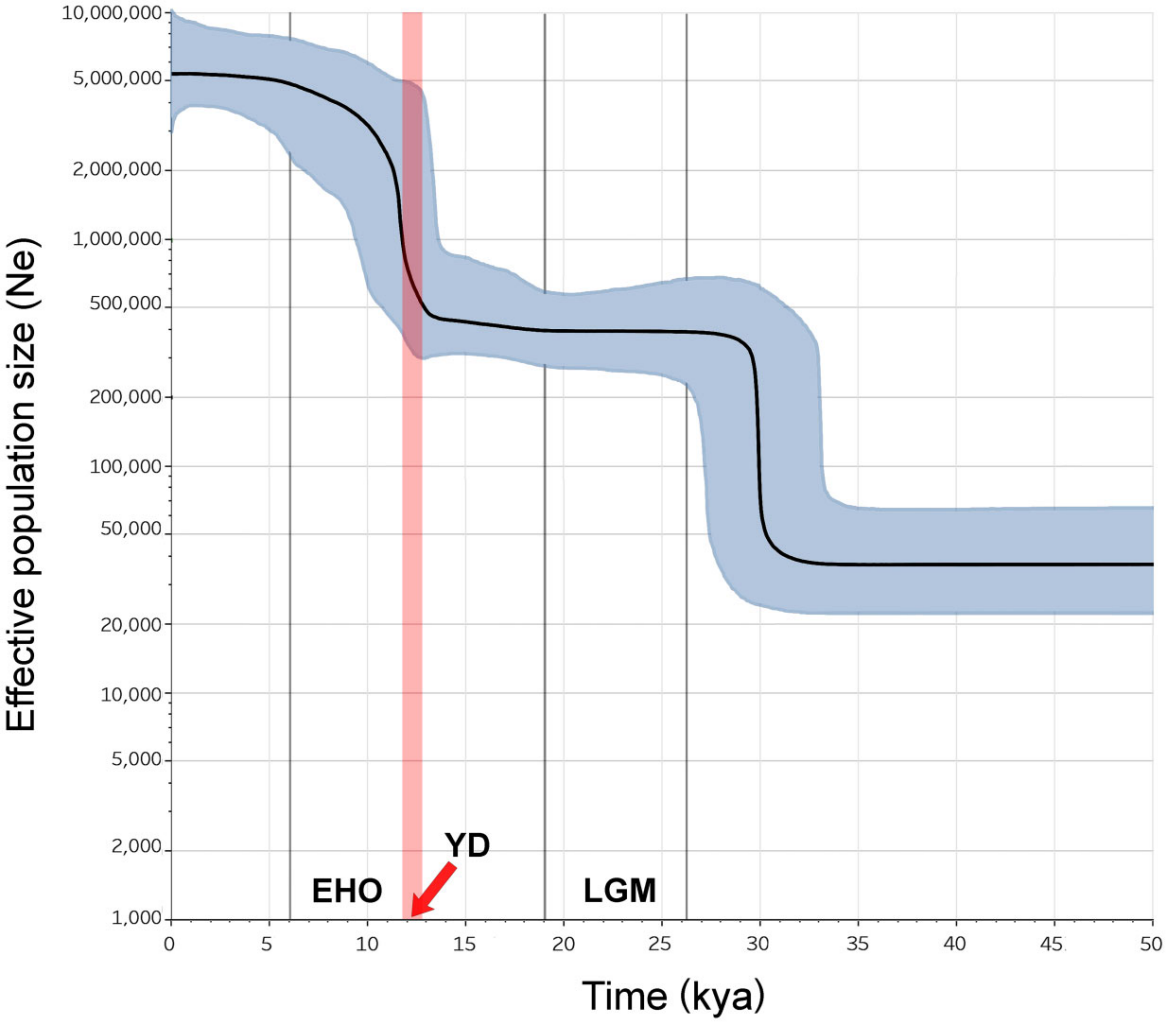
BSP of haplogroup A mitogenomes. The plot considers the 388 haplogroup A mitogenomes listed in supplementary table S1, Supplementary Material online. These include all *H. r. rustica* mitogenomes and 92% of those from *H. r. transitiva*. The black line indicates the median estimate of the effective population size and the blue shading shows the 95% highest posterior density limits. The time axis is limited to 50 kya, beyond which the curve remains flat.

The Bayesian skyline plot of fig. 4 shows changes in the effective population size over time for haplogroup A, which is typical of western Eurasia and by far the most represented in our survey encompassing all *H. r. rustica* mitogenomes and 92% of those from *H. r. transitiva*. It underwent two population growth events. The first, very sharp increase occurred ∼30 kya, prior to the last glacial maximum (LGM). This was followed by a plateau throughout the LGM and up to the YD (12.9–11.7 kya), when the second increase began, lasting until the end of the Early Holocene Optimum (EHO) ∼6 kya (Baker et al. 2017).

Population expansion has been documented during postglacial periods of many other bird populations, in parallel with and thanks to their northward range expansion (Milá et al. 2006; Zink and Gardner 2017). Migratory behavior might have both resulted from and played a role in this population expansion. Glacial cycles act as switches for the evolutionarily labile migratory behavior. Lacking a suitable habitat, species would retreat to their wintering ranges during glacial maxima and revert back to long-distance migration during interglacial periods (Zink and Gardner 2017). Our results on haplogroup A mitogenomes are consistent with *H. r. rustica* ancestors expanding northward from the eastern Mediterranean basin, which might have acted as a refugium during the LGM and the YD. *H. r. transitiva* would then have mainly derived from specimens/populations that maintained their sedentary behavior, while *H. r. rustica* would descend from those that differentiated and re-acquired a long-distance migratory behavior while expanding northward at the end (∼11.7 kya) of the YD. These climatic changes, and possibly the increase in energy consumption associated with the re-acquisition of the long-distance migratory behavior, appear to strongly affect the extent to which selection modulates the evolution of mitochondrial PCGs (supplementary fig. S4, Supplementary Material online). Taking the end of the YD (∼11.7 kya) as a cut-off in the phylogeny, it is evident that the ratio of divergence at non-synonymous and synonymous sites (dN/dS) is much higher when considering only variants accumulated after the YD (0.19 vs. 0.08, Fisher exact test *P-value* = 0.0001). This is particularly evident for genes encoding subunits of OXPHOS complexes I and V, thus supporting scenarios linking heat production and avian flight ability with mitogenome variation (Shen et al. 2009; Zhong et al. 2020).

Such a scenario would explain the sharing of haplogroup A by *rustica* and *transitiva* and many of its sub-branches and the “intermingling” of their haplotypes within these clades (fig. 2). However, it would also explain the detection of A sub-haplogroups within localized populations in Europe (*H. r. rustica*). The oldest are in the Mediterranean area, A1a2g in Italy and A1a1b3b in Spain (fig. 2), with ages of 11.6 ky and 11.4 ky, respectively. Thus, they arose shortly after the end of the YD. In contrast, the population-specific sub-haplogroups detected further north in Europe arose later with a south to north time profile: A1a2d1 in Switzerland (∼8.0 ky), A1a2e1a2a2 in Ukraine (∼7.6 ky) and A1a2e1a1a2a1b in Poland (∼6.0 ky). Their ages suggest that they arose *in situ* when these different European regions became suitable as nesting grounds. There is also a Levantine counterpart to the European-specific sub-haplogroups. This is represented by A1a2e1a3, the oldest *transitiva*-specific sub-haplogroup, which is dated at ∼11.6 kya, again immediately after the YD, underscoring its role in the differentiation of *rustica* and *transitiva*.

The chronological gradient from south to north suggests a history of northward expansion from the Near East or the Mediterranean basin at large. This is supported by the negative correlation between nucleotide diversity and latitude that we observed in *H. r. rustica* and *H. r. transitiva* populations, which encompass most of our dataset and were more densely sampled, when they were grouped into the following macro-groups: South (Algeria, Spain, South Italy and Israel), Center (North Italy and Switzerland), and North (Poland, Ukraine and Denmark) (supplementary fig. S7, Supplementary Material online). For haplogroup A1, a correlation (*P*-value <0.05) close to 1 was detected, thus confirming the overall reduction of mitogenome variation from South to North, as expected in models envisioning a more recent origin of central and northern European populations.

Recolonization of Europe from refugia following glacial retreat has been documented in a variety of species (Hewitt 1999, 2000; Hansson et al. 2008). The pattern of ever younger population-specific sub-haplogroups suggests a post-glacial expansion without major loss of genetic diversity and supports a relatively slow northward spreading—the so-called “phalanx-model” of colonization, as opposed to a “pioneer model” (Nichols and Hewitt 1994; Excoffier et al. 2009). Such a slow expansion is a feature of species with short dispersal, strict requirements for habitat, and/or dependency on other species (Hewitt 2004). Barn swallows preferentially nest in human structures and are closely associated with human agriculture. Their association with slow-moving agriculturalists might explain the age gradient from south to north Europe that we observed for the population-specific subclades. The first evidence of human built structures dates to around 12–15 kya (Potts 2012), and the expansion of agriculture from the Middle East might have begun as early as 12–13 kya (Salamini et al. 2002; Arranz-Otaegui et al. 2016). Thus, the second population increase is compatible with a role for rising temperatures at the beginning of the Holocene ∼12 kya (Taberlet et al. 1998) as well as for the association with human settlements (Smith et al. 2018).

### On the Origin of Barn Swallows

Previous comparisons of mtDNA variation in barn swallows, along with that seen in their closest relatives (Dor et al. 2010; Carter et al. 2020), have suggested that the ancestor of all *Hirundo* species most likely originated in Africa, as most of them have African distributions, including *H. aethiopica* and *H. angolensis* that are the closest relatives to *H. rustica* (Carter et al. 2020).

We further assessed this issue by adding the mitogenomes from other *Hirundo* species to the phylogeny of our barn swallows. The combined tree confirms that the closest species are all from Africa (*H. aethiopica, H. angolensis, H. nigrita, H. smithii*, and *H. albigularis*), thus supporting Africa as the ancestral continental source of *H. rustica*, and dates the divergence between *H. rustica* and *H. aethiopica,* the closest species, at about 493 kya (supplementary fig. S8, Supplementary Material online). However, it is possible that, when the HrAM arose ∼280 kya, the *H. rustica* population from which all modern *H. rustica* mitogenomes derive had already left Africa and entered into Eurasia.

## Conclusions

Over the course of years, numerous studies have shown that the information contained in mtDNA is phylogenetically best exploited when the sequence variation of the entire (or almost entire) mitogenome is assessed and the sequencing survey is carried out on numerous specimens sampled throughout the species distribution range. Here we employed this “magnifying glass” approach to reconstruct the genetic history of an iconic species, the barn swallow. Mitogenome data allowed us to build a detailed phylogeny for the species, to determine its coalescence time as well as the ages of its haplogroups, and to better define the matrilineal relationships between subspecies.

According to our data, the female barn swallow carrying HrAM lived 276.9 ± 24.3 kya, which is much earlier than previously thought (Zink et al. 2006; Dor et al. 2010; Scordato et al. 2017). Considering that, due to its reduced effective population size, mtDNA is much more prone to lineage loss and founder events than its autosomal counterpart, an almost 300 ky of mtDNA divergence implies an even older divergence time for most nuclear genes. This allows for plenty of polymorphism in the species complex and, probably, a rather extensive differentiation of its nuclear genome, thus explaining the observed flexibility and adaptability.

**Fig. 5.**
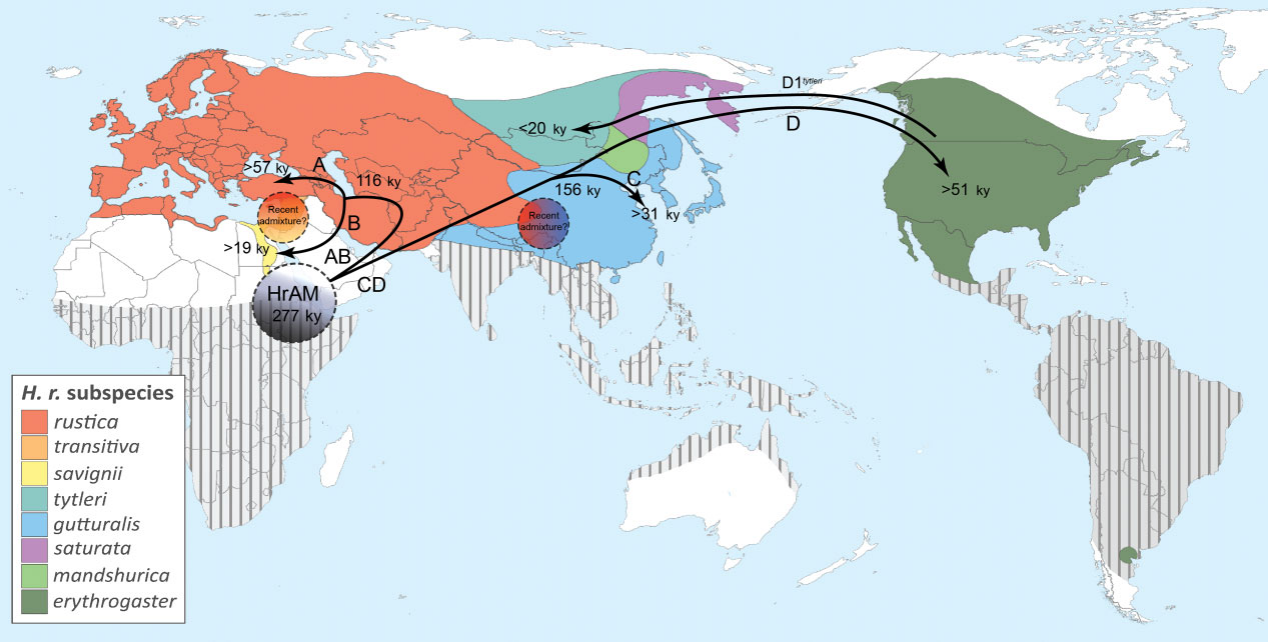
A model for the geographical and temporal spread of barn swallows. Map showing time divergence and hypothetical splits and diffusion routes of barn swallow haplogroups prior to the Younger Dryas and the subsequent climatic changes. The dashed grey circle indicates the possible homeland of the *Hirundo rustica* ancestral mitogenome (HrAM), while the other dashed circles indicate zones where two haplogroups are currently found, possibly indicating recent admixture between subspecies. Colors indicate the breeding ranges of the eight postulated barn swallow subspecies, while striped areas indicate wintering ranges (modified from Turner 2006).

In most cases, we observed complete subspecies and geographic specificity of mitogenome haplogroups, arising at different times and different places, which we employed together with estimated radiation times to postulate an overall model for the geographical and temporal spread of barn swallows (fig. 5). According to the mtDNA data, this species left Africa (or its vicinity) almost 300 kya, expanded first into Eurasia and then, at least 51 kya, into the Americas, from where a relatively recent (<20 kya) back migration to Asia took place. Subspecies differentiation occurred in parallel to the species dispersal, usually much earlier than previously suggested (Smith et al. 2018).

The notable exception to the haplogroup subspecies specificity is represented by the sedentary Levantine *H. r. transitiva* that extensively shares haplogroup A with the migratory *H. r. rustica* and haplogroup B, to a lesser extent, with the Egyptian *H. r. savignii*. We propose that *rustica* and *transitiva* derive from the same population source, which was located in the Levant and had adapted to sedentarism during the LGM. Our data indicate that the two subspecies began to split rather recently, shortly after 11.7 kya at the end of the YD. *H. r. rustica* would descend from individuals that re-acquired the long-distance migratory behavior while expanding northward to regions that were then becoming suitable as nesting grounds. In contrast, *H. r. transitiva* would derive from the Levantine component that remained *in situ* and maintained its sedentary behavior. Since then, however, *transitiva* did not remain genetically isolated, receiving genetic inputs and admixing with migratory *rustica* populations, as shown by the absence of significant correlations between genetic and geographic distances when assessing the shared haplogroups (supplementary fig. S9, Supplementary Material online), as well as the adjacent *savignii*.

This scenario, which is compatible with the presence of some haplogroup B mitogenomes in *transitiva* as well as its behavioral phenotype, is also supported by field and phenotypical observations (Ambrosini et al. 2009; Reiner Brodetzki et al. 2021), including the expression in *H. r. transitiva* of both elongated tail streamers and dark ventral coloration. The first feature is shared with *rustica*, but not its function as a sexual signal, and the second is shared with *savignii* (Vortman et al. 2011). Genetic admixture is also a plausible explanation for the detection of haplogroup A in one of the five *H. r. gutturalis* specimens from China.

Finally, *Hirundo rustica* has been strongly affected by climatic changes in the past. At the beginning of the Holocene, its population size began to grow extensively in parallel with temperature increases, and this growth was probably facilitated by the concomitant spread of agriculture and human built structures. It is also evident that climatic changes occurring during the LGM and the YD, and the possible resulting changes in migratory behavior, significantly affected the extent to which selection modulates gene sequence evolution, to a degree that is comparable with that reported in Neolithic animal domestication (Colli et al. 2015). The strong link of this widespread species with climate fluctuations and human activities makes it an excellent indicator for monitoring and assessing the impact of current global changes on wildlife.

## Materials and Methods

### Samples Analysed for Mitogenome Variation

We completely sequenced a total of 410 barn swallow mitogenomes. An additional *H. r. rustica* sample from Italy (no. 151) was extrapolated from Formenti et al. (2019). Samples were collected from Europe and Algeria (*n* = 340; *H. r. rustica*), Israel (*n* = 50; *H. r. transitiva*), China (*n* = 5; *H. r. gutturalis*), and the USA (Nebraska) (*n* = 15; *H. r. erythrogaster*). *H. r. rustica* were sampled in Denmark (*n* = 31), Italy (*n* = 171), Poland (*n* = 35), Spain (*n* = 31), Switzerland (*n* = 20), Ukraine (*n* = 20), and Algeria (*n* = 32), (supplementary fig. S2, Supplementary Material online). We extracted DNA either from tissues (muscle or liver) of specimens found dead (nos. 1, 20, 35, 58, 136, 302) or blood (all remaining samples). We obtained blood samples, under license according to national guidelines and legislation, by capturing individuals with mist-nets at the barns and cowsheds where barn swallows spend the nights during breeding. Venipuncture of the brachial vein, a minimally invasive technique (Arctander 1988), was performed to draw blood. Blood samples were collected and stored either in heparinized glass capillaries or dehydrated in ethanol. Blood samples from Spain and Nebraska arrived in Sodium-EDTA buffer. Detailed information on the barn swallow samples and their mitogenomes is provided in supplementary table S1, Supplementary Material online.

At the time when the analyses were performed, there were nine entire *H. rustica* mitogenomes in GenBank. However, none of these could be included in our analyses of entire mitogenomes due to the following sequence issues: KX398931 (*H. r. erythrogaster,*  Keepers et al. 2016, direct submission), a small nuclear mitochondrial (NUMT) sequence in *ND4*; MN356225 (*H. r. erythrogaster*, Feng et al. 2020, lack of *CR1*, *MP*, *ND6*, and *ME*; MN843972 (*H. r. tytleri*), MN829439 (*H. r. rustica*), MN830163 (*H. r. savignii*), MN954681, and MN840495 (*H. r. transitiva*), presence of NUMTs in *ND5* (Carter et al. 2020); MN909724 (*H. r. gutturalis*, Thirouin et al. 2020, direct submission), large insertion in *RNR2*, many gaps throughout the sequence, two large NUMTs in *ND3* and *ND5*; and KP148840 (*H. r. gutturalis*, Liu et al. 2015, direct submission), numerous NUMTs throughout the sequence. In addition, we extracted 16 low-coverage mitogenomes (eight from *H. r. savignii* and eight from *H. r. erythrogaster)* from the PRJNA323498 BioProject (Smith et al. 2018). They also harbored many gaps. However, most of these samples (*n* = 22) could be employed in the phylogenetic analyses of *ND2* and *CYB* gene sequences (see below).

### DNA Extraction

We obtained genomic DNA extracted from muscle or liver with the ReliaPrep™ (Promega Madison, WI, USA) gDNA Tissue kit, using the standard protocol for animal tissue. We extracted and purified blood samples using phenol-chloroform. These samples were prepared by breaking 1–2 cm of the glass capillary containing the blood (∼4 μl) and placing it overnight at 56°C in a 2 ml tube containing: lysis buffer B (400 mM Tris-HCl, pH 8.0; 100 mM EthyleneDiamine Tetra-Acetic acid [EDTA], pH 8.0; 1% Sodium Dodecyl Sulfate [SDS]), 250 µl of TBS buffer (20 mM Tris-HCl, pH 7.5; 150 mM NaCl), and 40 µl of Proteinase K (20 mg/ml). Samples from Spain and Nebraska were instead extracted with magnetic beads on a 16 Maxwell® RSC 16 instrument using the dedicated Blood DNA Kit (Promega) and employing the “Blood DNA” protocol. Sample preparation in this case was performed by adding 1–2 μl of blood to 300 μl of lysis buffer and 30 μl Proteinase K and incubating overnight at 56°C. Genomic DNAs were eluted into TE buffer (10 mM Tris-Cl, 1 mM EDTA) or elution buffer (Promega).

### Sanger Sequencing

The first five barn swallow mitogenomes that we obtained (nos. 1, 20, 35, 136, and 302) were Sanger sequenced. We designed an initial set of oligonucleotide pairs (not shown) using partial sequences of *H. r. rustica* mtDNA genes (mainly *RNR1, RNR2*, *ND2, CO1*, and *CYB*) available in GenBank using the Primer3Plus software (Untergasser et al. 2012) (https://www.primer3plus.com/). Then, by using a primer walking approach, we designed a set of oligonucleotide pairs that allowed the amplification of the entire mitogenome in 11 overlapping Polymerase Chain Reaction (PCR) fragments (supplementary table S5, Supplementary Material online) and a set of 33 additional oligonucleotides for sequencing (supplementary table S6, Supplementary Material online). This allowed the amplification and sequencing of mitogenome no. 20, our first *H. r. rustica* complete mitogenome. The other four mitogenomes (nos. 1, 35, 136, and 302) were then obtained by carrying out PCR reactions with a standard reaction mix (25 µl) containing 1X Buffer (1.5 mM MgCl2), 0.2 mM of each dNTP, 0.6U of GoTaq G2 Polymerase (Promega), 0.2 µM of each primer, and 20–30 ng of DNA, using the following PCR conditions: 94°C (2 min); 35 cycles of 94°C (30 s), 55°C (30 s), 72°C (2 min), and a final extension of 72°C (10 min). PCR products were visualized on a 1% agarose gel and amplicons were sequenced with standard dideoxy sequencing using BigDye v3.1 Chemistry (Applied Biosystems) on 3730xl and 3130xl Genetic Analyzer (Applied Biosystems) following the manufacturer’s protocol. Mitogenome no. 20 (MZ905359) was employed as the HrrRS. We then obtained the locus map of the barn swallow (supplementary fig. S1, Supplementary Material online) using the CGview Server (Grant and Stothard 2008).

### NGS Sequencing

We obtained 405 additional barn swallow mitogenomes by NGS sequencing and extrapolated one more from Formenti et al. (2019). We designed a set of three oligonucleotide pairs with similar T_m_ (∼60°C) and length (20 nt) (supplementary table S7, Supplementary Material online) to amplify the entire mtDNA molecule in three overlapping, long range PCR fragments of comparable lengths (∼6400 bps). Each fragment overlapped the next one by about 300–500 bps. PCRs were carried out in 50 µl reaction mix containing 1x GoTaq® Long PCR Master Mix (Promega), 0.3 μM of each primer and ∼150 ng of DNA template, using the following 2 step PCR thermal profile: 94°C (2 min); 20 cycles at 94°C (30 s), 58°C (30 s), 65°C (7 min), followed by 10 cycles at 94°C (30 s), 55°C (30 s), 65°C (7 min), and a final extension at 72°C (10 min). PCR products were checked by electrophoresis on 1% agarose gels. PCR purification was performed with the membrane method, Presto™ 96 Well PCR Cleanup Kit (Geneaid). Amplicons were quantified with the Quantus™ fluorometer (Promega) using the QuantiFluor® ONE dsDNA system. One nanogram for each of the three amplicons were combined for library preparation. The sequencing library was prepared with the Nextera™ DNA Flex Library Prep, following the manufacturer’s protocol steps: tagmentation of input DNA, amplification of tagmented DNA with the addition of pre-mixed dual-indexed adapters (IDT® for Illumina Nextera UD indexes or Nextera™ DNA CD Indexes), and PCR clean-up. Libraries were then checked on a 2% agarose gel, quantified using the Quantus™ fluorometer (Promega), normalized and pooled together. We then run the pooled normalized library on the 4150 TapeStation System (Agilent) and diluted to 4 nM using RSB resuspension buffer. Five microliter of pooled libraries were denatured using 5 μl of freshly prepared NaOH (0.2N) and diluted to the loading concentration of 6 pM (600 μl final volume) using HT1 hybridization buffer. This was finally sequenced on a MiSeq system (Illumina) using paired-end sequencing either with the MiSeq Reagent Kit v2, (2 × 150 or 2 × 250 cycles) or the MiSeq Reagent Kit v2 Nano (2 × 150 cycles). We also NGS sequenced mitogenome no. 20 (HrrRS) and the other four samples already Sanger sequenced (nos. 1, 35, 136 and 302), fully confirming the initial Sanger sequences.

### Analysis of Mitogenome Sequence Data

The raw MiSeq sequencer data were output in BCL format, demultiplexed, and converted to FASTQ format with the Illumina® bash package, bcl2fastq2 Conversion Software v2.20, and trimmed with Trim Galore! v 0.6.4 (Krueger 2012) to remove low-quality reads and adapters. We checked the quality of paired end reads by using FastQC v 0.11.9 (Andrews 2010). Files were subsequently converted from FASTQ to BAM by aligning and mapping the reads to the mitogenome no. 20 (HrrRS) using BWA v0.7.17 (Li 2013, direct submission). BAM files were analysed with Geneious 8.1.5 (Biomatters, Kearse et al. 2012). Variant calling was performed by setting the threshold for heteroplasmies at 30% of reads and considering only mutations in the range 30%–70% as heteroplasmic in phylogenetic analyses. Mitogenomes employed in the phylogenetic analyses were completely sequenced with an average depth of 459X. Finally, samples were exported in the standard FASTA format.

### Phylogenetic Analyses and Age Estimates of MtDNA Haplogroups

We built a maximum parsimony (MP) tree by manually programming the software mtPhyl v 5.003 (Eltsov and Volodko 2014) for the analysis of barn swallow mitogenomes (the modified.txt files are available on request). By comparing the mtDNA FASTA sequences to the HrrRS, the software allowed for the reconstruction of a coding-region (15,601 bps; nps 1–14,859, nps 16,068–16,740, nps 18,075–18,143) MP tree with detailed information concerning mutations (supplementary fig. S5, Supplementary Material online). We did not consider indels for tree construction. The tree was rooted using the available *Hirundo angolensis* (NC_050287) and *Hirundo aethiopica* (NC_050293) reference mitogenomes, and its topology was also confirmed by using the software Molecular Evolutionary Genetics Analysis X (MEGAX) (figs. 1 and 2) (Kumar et al. 2018).

We estimated ML and Bayesian ages of haplogroups and sub-haplogroups by using all barn swallow (*n* = 411) coding-region sequences (15,601 bps). We performed ML estimations using the BaseML package present in the PAMLX 1.3.1 software (Xu and Yang 2013) assuming the HKY85 (Hasegawa et al. 1985) mutation model with gamma-distributed (32 categories) rates (plus invariant sites) and 17 partitions (13 for protein coding genes, 1 for tRNAs, 1 for each rRNA gene, and 1 for intergenic regions) using the predefined tree obtained by the MP approach. We converted ML mutational distances into years by assuming an estimated split time between *Progne* (not shown) and *Hirundo* at 9.34 Mya (95% CI: 5.8–13.2 Mya) (Moyle et al. 2016), thus employing the standard approach that does not include the error on the calibration point.

We performed Bayesian estimations using the software Bayesian Evolutionary Analysis Sampling Trees (BEAST) 2.6.0 (Bouckaert et al. 2019) under the HKY substitution model (gamma-distributed rates plus invariant sites) with a relaxed clock (log normal). We entered as prior the clock value of 2.45 × 10^−8^ base substitution per nucleotide per year (or one mutation every 2616 years), derived from the rate calculated in the ML method. The chain length was established at 100,000,000 iterations, with samples drawn every 1,000 Markov chain Monte Carlo steps after a discarded burn-in of 10,000,000 steps (Olivieri et al. 2017). We performed the demographic analysis on the BEAST results using Tracer v1.7.1 (Rambaut et al. 2018) and Excel using a generation time of one year. We constructed Bayesian skyline plots (BSPs) using the output tree file and a stepwise constant.

To assess the subspecies specificity of major haplogroups in a wider sample encompassing also previously published sequences, we built a MP tree based on *ND2* (1,017 bps; nps 3980–4996) and *CYB* (1,058 bps; nps 13696–14753) gene sequences (fig. 3), for a total of 2075 bps. We aligned sequences with MEGAX and rooted the tree using the *H. aethiopica* (NC_050293) and *H. angolensis* (NC_050287) reference mitogenomes.

### Mitogenome Diversity and Latitude

To identify a potential correlation between mitogenome diversity (entire mitogenomes) and latitude, we measured both haplotype diversity (HD) and nucleotide diversity (Pi) for the most represented haplogroups (A1 as a whole as well as its major sub-haplogroups A1a1 and A1a2) using DNAsp 6.12.03 (Rozas et al. 2017). Both indices were compared with the average of the latitudes among the samples of each population using the software Tableau 2021.4 (https://www.tableau.com/).

### Isolation by Distance

To assess isolation by distance (IBD), genetic distance matrices were created based on PhiSt, which was computed using the R package *haplotypes* (Aktas 2020), while pairwise geographic distances were calculated with the *geodist* R package (Padgham and Sumner 2021), applying the geodesic methods provided in (Karney 2013). We tested the correlations between genetic and geographic matrices for the most represented haplogroups (A1 as a whole as well as its major sub-haplogroups A1a1 and A1a2) using a Mantel test and simple linear regression, to also account for possible type II errors (Teske et al. 2018). The Mantel test was performed using the R package *ade4* (Dray and Dufour 2007) with 999 permutations. This program tests for significant IBD by comparing the observed correlation with a histogram of simulated correlation categories and their frequency under the assumption of no IBD. Simple linear regression was performed in the *stats* package (R Core Team 2021). We generated plots in R using the package *ggplot2* (Wickham 2016).

## Supplementary Material

msac113_Supplementary_Data

## Data Availability

The sequence data for the 411 *H. rustica* mitogenomes are available in GenBank under accession numbers MZ905359, OK539050-OK539458, and OK624420. The raw NGS sequence data (fastq and bam files) are available under the ENA accession number PRJEB51610.

## References

[msac113-B1] Achilli A, Olivieri A, Pellecchia M, Uboldi C, Colli L, Al-Zahery N, Accetturo M, Pala M, Hooshiar Kashani B, Perego UA, et al 2008. Mitochondrial genomes of extinct aurochs survive in domestic cattle. Curr Biol. 18(4):R157–R158. 18302915 10.1016/j.cub.2008.01.019

[msac113-B2] Achilli A, Olivieri A, Soares P, Lancioni H, Hooshiar Kashani B, Perego UA, Nergadze SG, Carossa V, Santagostino M, Capomaccio S, et al 2012. Mitochondrial genomes from modern horses reveal the major haplogroups that underwent domestication. Proc Natl Acad Sci USA. 109(7):2449–2454. 22308342 10.1073/pnas.1111637109 PMC3289334

[msac113-B3] Aktas C . 2020. haplotypes: Manipulating DNA Sequences and Estimating Unambiguous Haplotype Network with Statistical Parsimony. R package version 1.1.2. https://CRAN.R-project.org/package=haplotypes.

[msac113-B4] Ambrosini R, Møller AP, Saino N. 2009. A quantitative measure of migratory connectivity. J Theor Biol. 257(2):203–211. 19108778 10.1016/j.jtbi.2008.11.019

[msac113-B5] Ambrosini R, Rubolini D, Trovò P, Liberini G, Bandini M, Romano A, Sicurella B, Scandolara C, Romano M, Saino N. 2012. Maintenance of livestock farming may buffer population decline of the Barn Swallow *Hirundo rustica*. Bird Conserv Int. 22:411–428.

[msac113-B6] Andrews S . 2010. FastQC a quality control tool for high throughput sequence data. Babraham bioinformatics. URL https://www.bioinformatics.babraham.ac.uk/projects/fastqc/.

[msac113-B7] Arctander P . 1988. Comparative studies of avian DNA by restriction fragment length polymorphism analysis: convenient procedures based on blood samples from live birds. J Ornithol. 129:205–216.

[msac113-B8] Areta JI, Salvador SA, Gandoy FA, Bridge ES, Gorleri FC, Pegan TM, Gulson-Castillo ER, Hobson KA, Winkler DW. 2021. Rapid adjustments of migration and life history in hemisphere-switching cliff swallows. Curr Biol. 31(13):2914–2919.e2. 33951458 10.1016/j.cub.2021.04.019

[msac113-B9] Arranz-Otaegui A, Colledge S, Zapata L, Teira-Mayolini LC, Ibáñez JJ. 2016. Regional diversity on the timing for the initial appearance of cereal cultivation and domestication in southwest Asia. Proc Natl Acad Sci USA. 113(49):14001–14006. 27930348 10.1073/pnas.1612797113 PMC5150421

[msac113-B10] Baker JL, Lachniet MS, Chervyatsova O, Asmerom Y, Polyak VJ. 2017. Holocene warming in western continental Eurasia driven by glacial retreat and greenhouse forcing. Nat Geosci. 10:430–435.

[msac113-B11] Balbontín J, Møller AP, Hermosell IG, Marzal A, Reviriego M, de Lope F. 2009. Geographic patterns of natal dispersal in barn swallows *Hirundo rustica* from Denmark and Spain. Behav Ecol Sociobiol. 63:1197–1205.

[msac113-B12] Barth JMI, Damerau M, Matschiner M, Jentoft S, Hanel R. 2017. Genomic differentiation and demographic histories of Atlantic and Indo-Pacific yellowfin tuna (*Thunnus albacares*) populations. Genome Biol Evol. 9(4):1084–1098. 28419285 10.1093/gbe/evx067 PMC5408087

[msac113-B13] Battaglia V, Gabrieli P, Brandini S, Capodiferro MR, Javier PA, Chen XG, Achilli A, Semino O, Gomulski LM, Malacrida AR, et al 2016. The worldwide spread of the tiger mosquito as revealed by mitogenome haplogroup diversity. Front Genet. 7:208. 27933090 10.3389/fgene.2016.00208 PMC5120106

[msac113-B14] Behar DM, van Oven M, Rosset S, Metspalu M, Loogväli EL, Silva NM, Kivisild T, Torroni A, Villems R. 2012. “A Copernican” reassessment of the human mitochondrial DNA tree from its root. Am J Hum Genet. 90(4):675–684. 22482806 10.1016/j.ajhg.2012.03.002 PMC3322232

[msac113-B15] Bouckaert R, Vaughan TG, Barido-Sottani J, Duchêne S, Fourment M, Gavryushkina A, Heled J, Jones G, Kühnert D, De Maio N, et al 2019. BEAST 2.5: an advanced software platform for Bayesian evolutionary analysis. PLoS Comp Biol. 15(4):e1006650. 10.1371/journal.pcbi.1006650 PMC6472827 30958812

[msac113-B16] Brown CR, Brown MB. 1999. Barn swallow (Hirundo rustica). Vol. 452. Philadelphia (PA): The birds of North America. Inc., p. 32.

[msac113-B17] Carter JK, Innes P, Goebl AM, Johnson B, Gebert M, Attia Z, Gabani Z, Li R, Melie T, Dart C, et al 2020. Complete mitochondrial genomes provide current refined phylogenomic hypotheses for relationships among ten *Hirundo* species. Mitochondrial DNA B Resour. 5(3):2881–2885. 33457987 10.1080/23802359.2020.1790999 PMC7783031

[msac113-B18] Cheng T . 1987. A synopsis to the avifauna of China. Hamburg: Science Press, Paul Parey. ISBN 10: 3490125185.

[msac113-B19] Cole TL, Ksepka DT, Mitchell KJ, Tennyson AJD, Thomas DB, Pan H, Zhang G, Rawlence NJ, Wood JR, Bover P, et al 2019. Mitogenomes uncover extinct penguin taxa and reveal island formation as a key driver of speciation. Mol Biol Evol. 36(4):784–797. 30722030 10.1093/molbev/msz017

[msac113-B20] Colli L, Lancioni H, Cardinali I, Olivieri A, Capodiferro MR, Pellecchia M, Rzepus M, Zamani W, Naderi S, Gandini F, et al 2015. Whole mitochondrial genomes unveil the impact of domestication on goat matrilineal variability. BMC Genomics. 16:1115. 26714643 10.1186/s12864-015-2342-2 PMC4696231

[msac113-B21] de Manuel M, Barnett R, Sandoval-Velasco M, Yamaguchi N, Garrett Vieira F, Zepeda Mendoza ML, Liu S, Martin MD, Sinding MS, Mak SST, et al 2020. The evolutionary history of extinct and living lions. Proc Natl Acad Sci USA. 117(20):10927–10934. 32366643 10.1073/pnas.1919423117 PMC7245068

[msac113-B22] Dickinson EC, Dekker RWRJ. 2001. Systematic notes on Asian birds. 13. A preliminary review of the Hirundinidae. Zool Verh. 335:127–144.

[msac113-B23] Dickinson EC, Eck S, Milensky CM. 2002. Systematic notes on Asian birds. 31. Eastern races of the barn swallow *Hirundo rustica* Linnaeus, 1758. Zool Verh. 340:201–203.

[msac113-B24] Dor R, Safran RJ, Sheldon FH, Winkler DW, Lovette IJ. 2010. Phylogeny of the genus *Hirundo* and the barn swallow subspecies complex. Mol Phylogenet Evol. 56(1):409–418. 20152914 10.1016/j.ympev.2010.02.008

[msac113-B25] Dor R, Safran RJ, Vortman Y, Lotem A, McGowan A, Evans MR, Lovette IJ. 2012. Population genetics and morphological comparisons of migratory European (*Hirundo rustica rustica*) and sedentary East-Mediterranean (*Hirundo rustica transitiva*) barn swallows. J Hered. 103(1):55–63. 22071313 10.1093/jhered/esr114

[msac113-B26] Dray S, Dufour A. 2007. The ade4 package: implementing the duality diagram for ecologists. J Stat Softw. 22(4):1–20.

[msac113-B27] Eltsov N, Volodko N. 2014. mtPhyl-software tool for human mtDNA analysis and phylogeny reconstruction. Available from: http://eltsov.org.

[msac113-B28] Excoffier L, Foll M, Petit RJ. 2009. Genetic consequences of range expansions. Annu Rev Ecol Evol Syst. 40:481–501.

[msac113-B29] Feng S, Stiller J, Deng Y, Armstrong J, Fang Q, Reeve AH, Xie D, Chen G, Guo C, Faircloth BC, et al 2020. Dense sampling of bird diversity increases power of comparative genomics. Nature. 587(7833):252–257. Erratum in: Nature. 2021; 592(7856):E24. 33177665 10.1038/s41586-020-2873-9 PMC7759463

[msac113-B30] Formenti G, Chiara M, Poveda L, Francoijs KJ, Bonisoli-Alquati A, Canova L, Gianfranceschi L, Horner DS, Saino N. 2019. SMRT long reads and direct label and stain optical maps allow the generation of a high-quality genome assembly for the European barn swallow (*Hirundo rustica rustica*). Gigascience. 8(1):giy142. 10.1093/gigascience/giy142 PMC6324554 30496513

[msac113-B31] Grant JR, Stothard P. 2008. The CGView server: a comparative genomics tool for circular genomes. Nucleic Acids Res. 36(Web Server issue):W181–W184. 18411202 10.1093/nar/gkn179 PMC2447734

[msac113-B32] Green A . 1988. Cultural responses to the migration of the barn swallow in Europe. In Stanley Cramp editor. Handbook of the birds of Europe, the Middle East and North Africa: the birds of the Western Palearctic, vol. 5. Oxford & New York: Oxford University Press, p. 263ff.

[msac113-B33] Hansson B, Hasselquist D, Tarka M, Zehtindjiev P, Bensch S. 2008. Postglacial colonisation patterns and the role of isolation and expansion in driving diversification in a passerine bird. PLoS One. 3:e2794. 18665223 10.1371/journal.pone.0002794 PMC2467487

[msac113-B34] Hasegawa M, Kishino H, Yano T. 1985. Dating of the human-ape splitting by a molecular clock of mitochondrial DNA. J Mol Evol. 22(2):160–174. 3934395 10.1007/BF02101694

[msac113-B35] Hewitt GM . 1999. Post-glacial re-colonization of European biota. Biol J Linn Soc. 68:87–112.

[msac113-B36] Hewitt GM . 2000. The genetic legacy of the Quaternary ice ages. Nature. 405:907–913. 10879524 10.1038/35016000

[msac113-B37] Hewitt GM . 2004. Genetic consequences of climatic oscillations in the Quaternary. Phil Trans R Soc B Biol Sci. 359:183–195. 10.1098/rstb.2003.1388 PMC1693318 15101575

[msac113-B38] Hobson KA, Kardynal KJ, Van Wilgenburg SL, Albrecht G, Salvadori A, Cadman MD, Liechti F, Fox JW. 2015. A continent-wide migratory divide in North American breeding barn swallows (*Hirundo rustica*). PLoS One. 10(6):e0129340. 26065914 10.1371/journal.pone.0129340 PMC4466147

[msac113-B39] Karney CFF . 2013. Algorithms for geodesics. J Geod. 87:43–55.

[msac113-B40] Kearse M, Moir R, Wilson A, Stones-Havas S, Cheung M, Sturrock S, Buxton S, Cooper A, Markowitz S, Duran C, et al 2012. Geneious Basic: an integrated and extendable desktop software platform for the organization and analysis of sequence data. Bioinformatics. 28(12):1647–1649. 22543367 10.1093/bioinformatics/bts199 PMC3371832

[msac113-B41] Keepers KG, Scordato ESC, Jenkins B, Safran RJ, Kane NC. 2016. The complete annotated mitochondrial genome of the North American barn swallow, Hirundo rustica erythrogaster. Direct submission.

[msac113-B42] Kiat Y, Izhaki I, Sapir N. 2019. The effects of long-distance migration on the evolution of moult strategies in Western-Palearctic passerines. Biol Rev. 94(2):700–720. 30334341 10.1111/brv.12474

[msac113-B43] Krueger F . 2012. Trim Galore: a wrapper tool around Cutadapt and FastQC to consistently apply quality and adapter trimming to FastQ files, with some extra functionality for MspI-digested RRBS-type (Reduced Representation Bisufite-Seq) libraries. Available from: http://www.bioinformatics.babraham.ac.uk/projects/trim_galore/.

[msac113-B44] Kumar S, Stecher G, Li M, Knyaz C, Tamura K. 2018. MEGA X: molecular evolutionary genetics analysis across computing platforms. Mol Biol Evol. 35(6):1547–1549. 29722887 10.1093/molbev/msy096 PMC5967553

[msac113-B45] Li H . 2013. Aligning sequence reads, clone sequences and assembly contigs with BWA-MEM. arXiv 2013/05/26. https://arxiv.org/abs/1303.3997v2.

[msac113-B46] Liechti F, Scandolara C, Rubolini D, Ambrosini R, Korner-Nievergelt F, Hahn S, Lardelli R, Romano M, Caprioli M, Romano A, et al 2015. Timing of migration and residence areas during the non-breeding period of barn swallows *Hirundo rustica* in relation to sex and population. J Avian Biol. 46(3):254–265.

[msac113-B47] Liu J, Liu S, Shao C, Zhang Y, Xie Y, Tang Q, Shen Y, Xie J. 2015. Characterization of the complete mitochondrial genome of *Hirundo rustica*. Direct submission.

[msac113-B48] Liu Y, Scordato ES, Zhang Z, Evans M, Safran RJ. 2020. Analysing phenotypic variation in barn swallows (*Hirundo rustica*) across China to assess subspecies status. Biol J Linn Soc. 131(2):319–331.

[msac113-B49] Mackiewicz P, Urantówka AD, Kroczak A, Mackiewicz D. 2019. Resolving phylogenetic relationships within passeriformes based on mitochondrial genes and inferring the evolution of their mitogenomes in terms of duplications. Genome Biol Evol. 11(10):2824–2849. 31580435 10.1093/gbe/evz209 PMC6795242

[msac113-B50] Malaitad T, Laipasu P, Eiamampai K, Poeaim S. 2016. Identification of the subspecies and gender of barn swallow (*Hirundo rustica*). Int J Agric Technol. 12.7.1:1549–1556.

[msac113-B51] Mead C . 2002. Barn swallow *Hirundo rustica*. In The migration atlas: movements of the birds of Britain and Ireland. London: T. & A.D. Poyser.

[msac113-B52] Miao YW, Peng MS, Wu GS, Ouyang YN, Yang ZY, Yu N, Liang JP, Pianchou G, Beja-Pereira A, Mitra B, et al 2013. Chicken domestication: an updated perspective based on mitochondrial genomes. Heredity (Edinb). 110(3):277–282. 23211792 10.1038/hdy.2012.83 PMC3668654

[msac113-B53] Milá B, Smith TB, Wayne RK. 2006. Postglacial population expansion drives the evolution of long-distance migration in a songbird. Evolution. 60:2403–2409. 17236431

[msac113-B54] Møller AP . 1994. Sexual selection and the barn swallow: Oxford University Press.

[msac113-B55] Møller AP . 2019. Parallel declines in abundance of insects and insectivorous birds in Denmark over 22 years. Ecol Evol. 9(11):6581–6587. 31236245 10.1002/ece3.5236 PMC6580276

[msac113-B56] Morin PA, Parsons KM, Archer FI, Ávila-Arcos MC, Barrett-Lennard LG, Dalla Rosa L, Duchêne S, Durban JW, Ellis GM, Ferguson SH, et al 2015. Geographic and temporal dynamics of a global radiation and diversification in the killer whale. Mol Ecol. 24(15):3964–3979. 26087773 10.1111/mec.13284

[msac113-B57] Moyle RG, Oliveros CH, Andersen MJ, Hosner PA, Benz BW, Manthey JD, Travers SL, Brown RM, Faircloth BC. 2016. Tectonic collision and uplift of Wallacea triggered the global songbird radiation. Nat Commun. 7:12709. 27575437 10.1038/ncomms12709 PMC5013600

[msac113-B58] Nichols RA, Hewitt GM. 1994. The genetic consequences of long-distance dispersal during colonization. Heredity. 72:312–317.

[msac113-B59] Niedziałkowska M, Tarnowska E, Ligmanowska J, Jędrzejewska B, Podgórski T, Radziszewska A, Ratajczyk I, Kusza S, Bunevich AN, Danila G, et al 2021. Clear phylogeographic pattern and genetic structure of wild boar *Sus scrofa* population in Central and Eastern Europe. Sci Rep. 11(1):9680. 33958636 10.1038/s41598-021-88991-1 PMC8102581

[msac113-B60] Olivieri A, Sidore C, Achilli A, Angius A, Posth C, Furtwängler A, Brandini S, Capodiferro MR, Gandini F, Zoledziewska M, et al 2017. Mitogenome diversity in Sardinians: A genetic window onto an island’s past. Mol Biol Evol. 34(5):1230–1239. 28177087 10.1093/molbev/msx082 PMC5400395

[msac113-B61] Padgham M, Sumner MD. 2021. geodist: Fast, Dependency-Free Geodesic Distance Calculations. R package version 0.0.7. https://CRAN.R-project.org/package=geodist.

[msac113-B62] Peng MS, Xu W, Song JJ, Chen X, Sulaiman X, Cai L, Liu HQ, Wu SF, Gao Y, Abdulloevich NT, et al 2018. Mitochondrial genomes uncover the maternal history of the Pamir populations. Eur J Hum Genet. 26(1):124–136. 29187735 10.1038/s41431-017-0028-8 PMC5839027

[msac113-B63] Potts R . 2012. Evolution and environmental change in early human prehistory. Annu Rev Anthropol. 41:151–167.

[msac113-B64] Quinn TW, Wilson AC. 1993. Sequence evolution in and around the mitochondrial control region in birds. J Mol Evol. 37(4):417–425. 8308909 10.1007/BF00178871

[msac113-B65] Rambaut A, Drummond AJ, Xie D, Baele G, Suchard MA. 2018. Posterior summarization in Bayesian phylogenetics using Tracer 1.7. Syst Biol. 67(5):901–904. 29718447 10.1093/sysbio/syy032 PMC6101584

[msac113-B66] Reiner Brodetzki T, Lotem A, Safran RJ, Hauber ME. 2021. Lack of subspecies-recognition in breeding barn swallows (*Hirundo rustica transitiva*). Behav Processes. 189:104422. 33992739 10.1016/j.beproc.2021.104422

[msac113-B67] Romano A, Saino N, Møller AP. 2017. Viability and expression of sexual ornaments in the barn swallow *Hirundo rustica*: a meta-analysis. J Evol Biol. 30(10):1929–1935. 28787531 10.1111/jeb.13151

[msac113-B68] Rozas J, Ferrer-Mata A, Sánchez-DelBarrio JC, Guirao-Rico S, Librado P, Ramos-Onsins SE, Sánchez-Gracia A. 2017. DnaSP 6: DNA sequence polymorphism analysis of large data sets. Mol Biol Evol. 34(12):3299–3302. 29029172 10.1093/molbev/msx248

[msac113-B69] Safran RJ, Scordato ES, Wilkins MR, Hubbard JK, Jenkins BR, Albrecht T, Flaxman SM, Karaardıç H, Vortman Y, Lotem A, et al 2016. Genome-wide differentiation in closely related populations: the roles of selection and geographic isolation. Mol Ecol. 25(16):3865–3883. 27357267 10.1111/mec.13740

[msac113-B70] Salamini F, Ozkan H, Brandolini A, Schäfer-Pregl R, Martin W. 2002. Genetics and geography of wild cereal domestication in the Near East. Nat Rev Genet. 3(6):429–441. 12042770 10.1038/nrg817

[msac113-B71] Santure AW, Ewen JG, Sicard D, Roff DA, Møller AP. 2010. Population structure in the barn swallow, *Hirundo rustica*: a comparison between neutral DNA markers and quantitative traits. Biol J Linn Soc. 99(2):306–314.

[msac113-B72] Scandolara C, Lardelli R, Sgarbi G, Caprioli M, Ambrosini R, Rubolini D, Saino N. 2014. Context-, phenotype-, and kin-dependent natal dispersal of barn swallows (*Hirundo rustica*). Behav Ecol. 25(1):180–190.

[msac113-B73] Scordato ESC, Smith CCR, Semenov GA, Liu Y, Wilkins MR, Liang W, Rubtsov A, Sundev G, Koyama K, Turbek SP, et al 2020. Migratory divides coincide with reproductive barriers across replicated avian hybrid zones above the Tibetan Plateau. Ecol Lett. 23(2):231–241. 31746098 10.1111/ele.13420

[msac113-B74] Scordato ESC, Wilkins MR, Semenov G, Rubtsov AS, Kane NC, Safran RJ. 2017. Genomic variation across two barn swallow hybrid zones reveals traits associated with divergence in sympatry and allopatry. Mol Ecol. 26(20):5676–5691. 28777875 10.1111/mec.14276

[msac113-B75] Sheldon FH, Whittingham LA, Moyle RG, Slikas B, Winkler DW. 2005. Phylogeny of swallows (Aves: Hirundinidae) estimated from nuclear and mitochondrial DNA sequences. Mol Phylogenet Evol. 35(1):254–270. 15737595 10.1016/j.ympev.2004.11.008

[msac113-B76] Shen YY, Shi P, Sun YB, Zhang YP. 2009. Relaxation of selective constraints on avian mitochondrial DNA following the degeneration of flight ability. Genome Res. 19(10):1760–1765. 19617397 10.1101/gr.093138.109 PMC2765268

[msac113-B77] Shirihai H, Dovrat E, Christie DA, Harris A. 1996. The birds of Israel. London: Academic Press.

[msac113-B78] Smith CCR, Flaxman SM, Scordato ESC, Kane NC, Hund AK, Sheta BM, Safran RJ. 2018. Demographic inference in barn swallows using whole-genome data shows signal for bottleneck and subspecies differentiation during the Holocene. Mol Ecol. 27(21):4200–4212. 30176075 10.1111/mec.14854

[msac113-B79] Stewart JB, Freyer C, Elson JL, Wredenberg A, Cansu Z, Trifunovic A, Larsson NG. 2008. Strong purifying selection in transmission of mammalian mitochondrial DNA. PLoS Biol. 6(1):e10. 18232733 10.1371/journal.pbio.0060010 PMC2214808

[msac113-B80] Taberlet P, Fumagalli L, Wust-Saucy AG, Cosson JF. 1998. Comparative phylogeography and postglacial colonization routes in Europe. Mol Ecol. 7(4):453–464. 9628000 10.1046/j.1365-294x.1998.00289.x

[msac113-B81] Teglhøj PG . 2020. Natal dispersal and recruitment of barn swallows *Hirundo rustica* in an urban habitat. Bird Study. 67(4):420–428.

[msac113-B82] Teske PR, Golla TR, Sandoval-Castillo J, Emami-Khoyi A, van der Lingen CD, von der Heyden S, Chiazzari B, Jansen van Vuuren B, Beheregaray LB. 2018. Mitochondrial DNA is unsuitable to test for isolation by distance. Sci Rep. 8(1):8448. 29855482 10.1038/s41598-018-25138-9 PMC5981212

[msac113-B83] Thirouin KR, Goebl AM, Carter J, Scordato E, Hund A, Safran RJ, KaneN. Ecol Evol Biol. 2020. Direct submission.

[msac113-B84] Torroni A, Achilli A, Macaulay V, Richards M, Bandelt HJ. 2006. Harvesting the fruit of the human mtDNA tree. Trends Genet. 22(6):339–345. 16678300 10.1016/j.tig.2006.04.001

[msac113-B85] Turner A . 2006. The barn swallow. London: T. & A.D. Poyser.

[msac113-B86] Turner A, Rose C. 2010. A handbook to the swallows and martins of the world. London: A & C Black.

[msac113-B87] Untergasser A, Cutcutache I, Koressaar T, Ye J, Faircloth BC, Remm M, Rozen SG. 2012. Primer3–new capabilities and interfaces. Nucleic Acids Res. 40(15):e115. 22730293 10.1093/nar/gks596 PMC3424584

[msac113-B88] Urantówka AD, Kroczak A, Mackiewicz P. 2020. New view on the organization and evolution of Palaeognathae mitogenomes poses the question on the ancestral gene rearrangement in Aves. BMC Genomics. 21(1):874. 33287726 10.1186/s12864-020-07284-5 PMC7720580

[msac113-B89] R Core Team . 2021. R: A language and environment for statistical computing. Vienna, Austria: R Foundation for Statistical Computing. URL https://www.R-project.org/.

[msac113-B90] von Rönn JA, Shafer AB, Wolf JB. 2016. Disruptive selection without genome-wide evolution across a migratory divide. Mol Ecol. 25(11):2529–2541. 26749140 10.1111/mec.13521

[msac113-B91] Vortman Y, Lotem A, Dor R, Lovette IJ, Safran RJ. 2011. The sexual signals of the East-Mediterranean barn swallow: a different swallow tale. Behavioral Ecol. 22(6):1344–1352.

[msac113-B92] Wickham H . 2016. ggplot2: elegant graphics for data analysis. New York (NY): Springer-Verlag.

[msac113-B93] Winkler DW, Gandoy FA, Areta JI, Iliff MJ, Rakhimberdiev E, Kardynal KJ, Hobson KA. 2017. Long-distance range expansion and rapid adjustment of migration in a newly established population of barn swallows breeding in Argentina. Curr Biol. 27(7):1080–1084. 28318974 10.1016/j.cub.2017.03.006

[msac113-B94] Xu B, Yang Z. 2013. PAMLX: a graphical user interface for PAML. Mol Biol Evol. 30(12):2723–2724. 24105918 10.1093/molbev/mst179

[msac113-B95] Zhong Y, Zhou M, Ouyang B, Zeng C, Zhang M, Yang J. 2020. Complete mtDNA genome of *Otus sunia* (Aves. Strigidae) and the relaxation of selective constrains on strigiformes mtDNA following evolution. Genomics. 112(5):3815–3825. 32135299 10.1016/j.ygeno.2020.02.018

[msac113-B96] Zink RM, Gardner AS. 2017. Glaciation as a migratory switch. Sci Adv. 3:e1603133. 28948216 10.1126/sciadv.1603133 PMC5606702

[msac113-B97] Zink RM, Pavlova A, Rohwer S, Drovetski SV. 2006. Barn swallows before barns: population histories and intercontinental colonization. Proc Biol Sci. 273(1591):1245–1251. 16720398 10.1098/rspb.2005.3414 PMC1560278

