# Supplementary material for: The Mitogenome Relationships and Phylogeography of Barn Swallows (Hirundo rustica)
Source: Mol Biol Evol. 2022 May 25;39(6):msac113. doi: 10.1093/molbev/msac113 (PMC9174979; doi:10.1093/molbev/msac113)
Supplement: msac113_Supplementary_Data [file msac113_supplementary_data.zip › Lombardo_et_al._2022_SupplementaryMaterial_May19_2022.pdf]

SUPPLEMENTARY MATERIAL

The Mitogenome Relationships and Phylogeography of Barn Swallows (*Hirundo rustica*)

Gianluca Lombardo,[1] Nicola Rambaldi Migliore,[1] Giulia Colombo,[1] Marco Rosario Capodiferro,[1] Giulio Formenti,[2] Manuela Caprioli,[3] Elisabetta Moroni,[1] Leonardo Caporali,[4] Hovirag Lancioni,[5] Simona Secomandi,[6] Guido Roberto Gallo,[6] Alessandra Costanzo,[3] Andrea Romano,[3] Maria Garofalo,[7] Cristina Cereda,[7] Valerio Carelli,[4,8] Lauren Gillespie,[9] Yang Liu,[10] Yosef Kiat,[11] Alfonso Marzal,[12] Cosme López-Calderón,[13] Javier Balbontín,[14] Timothy A. Mousseau,[15] Piotr Matyjasiak,[16] Anders Pape Møller,[17] Ornella Semino,[1] Roberto Ambrosini,[3] Andrea Bonisoli Alquati,[18] Diego Rubolini,[3] Luca Ferretti,[1] Alessandro Achilli,[1] Luca Gianfranceschi,[6] Anna Olivieri,[*,1] and Antonio Torroni[*,1]

[1] Dipartimento di Biologia e Biotecnologie "Lazzaro Spallanzani", Università di Pavia, 27100 Pavia, Italy
[2] Vertebrate Genome Laboratory, The Rockefeller University, New York, NY 10065, USA
[3] Dipartimento di Scienze e Politiche Ambientali, Università degli Studi di Milano, 20133 Milan, Italy
[4] IRCCS Istituto delle Scienze Neurologiche di Bologna, Programma di Neurogenetica, 40139 Bologna, Italy
[5] Dipartimento di Chimica, Biologia e Biotecnologie, Università di Perugia, 06123 Perugia, Italy
[6] Dipartimento di Bioscienze, Università degli Studi di Milano, 20133 Milan, Italy
[7] Genomic and Post-Genomic Unit, IRCCS Mondino Foundation, 27100 Pavia, Italy
[8] Dipartimento di Scienze Biomediche e Neuromotorie, Università di Bologna, 40139 Bologna, Italy
[9] Department of Academic Education, Central Community College, Columbus, NE 68601, USA
[10] State Key Laboratory of Biocontrol, School of Ecology, Sun Yat-sen University, Guangzhou 510275, China
[11] Israeli Bird Ringing Center (IBRC), Israel Ornithological Center, Tel Aviv, Israel
[12] Department of Zoology, University of Extremadura, 06071 Badajoz, Spain
[13] Department of Wetland Ecology, Estación Biológica de Doñana CSIC, 41092 Seville, Spain
[14] Department of Zoology, University of Seville, 41012 Seville, Spain
[15] Department of Biological Sciences, University of South Carolina, Columbia, SC 29208, USA
[16] Institute of Biological Sciences, Cardinal Stefan Wyszyński University in Warsaw, 01-938 Warsaw, Poland
[17] Ecologie Systématique Evolution, Université Paris-Sud, CNRS, AgroParisTech, Université Paris-Saclay, 91405, Orsay Cedex, France
[18] Department of Biological Sciences, California State Polytechnic University - Pomona, Pomona, CA 91767, USA

[*] Shared last authors and **corresponding authors:** E-mails: antonio.torroni@unipv.it; anna.olivieri@unipv.it

SUPPLEMENTARY FIGURES

Fig. S1. Map of the *Hirundo rustica rustica* mitogenome. This map refers to the reference sequence (HrrRS, mitogenome #20, MZ905359) obtained with the CGview Server (Grant and Stothard 2008). Genes are represented as blocks of different colours. PCG indicates the 13 protein-coding genes. tRNA genes are labelled according to single-letter abbreviations. Direction of gene transcription is shown by arrows. The GC content is plotted using a black sliding window, as the deviation from the average GC content of the entire sequence. Positive and negative GC skews are relative to the average GC content of the entire sequence. CR1 is 1208 bps in length, and maps between the MT-TT and MT-TP genes. Ten conserved sequence blocks (CSBs) within CR1 were identified by alignment with other avian sequences (Eberhard et al. 2001; Pereira et al. 2004). These include the CSB1-like, F, E, D, C, B-box (B2) of Saunders and Edwards (2000), and the Bird, B, CSB1 and CSB2/3 boxes plus the c-string (Goose hairpin, Quinn and Wilson 1993). CR2, which derives from a duplication of CR1 (Urantówka et al. 2020) and maps between the MT-TE and MT-TF genes, is larger (1334 bps) because of a longer microsatellite (36 CAAAAA repeats instead of 15) at the 3' end. The gene organization corresponds to that previously reported for all other Hirundinidae with eight tRNA genes and the *ND6* gene located on the light strand. The heavy strand base composition comprises A-5688 (31.4%), T-4305 (23.7%), C-5520 (30.4%) and G-2630 (14.5%), with an A+T (55.1%) content higher than the G+C content as reported in other Hirundinidae (Cerasale et al. 2012; Liu et al. 2016; Carter et al. 2020).

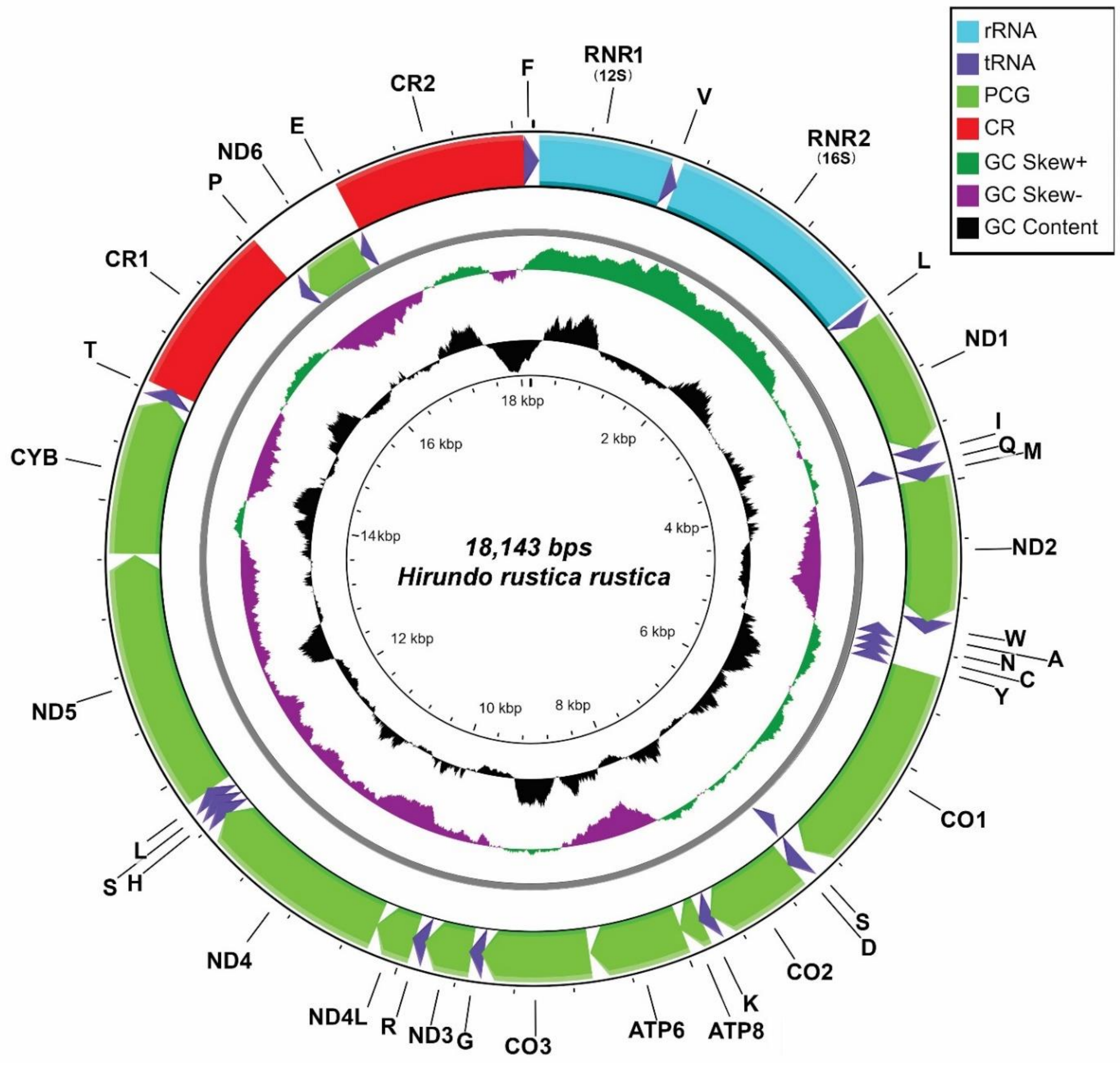

Fig. S2. **Sampling locations of *Hirundo rustica* mitogenomes.** Geographical sampling locations of the 411 barn swallows analysed for mtDNA sequence variation.

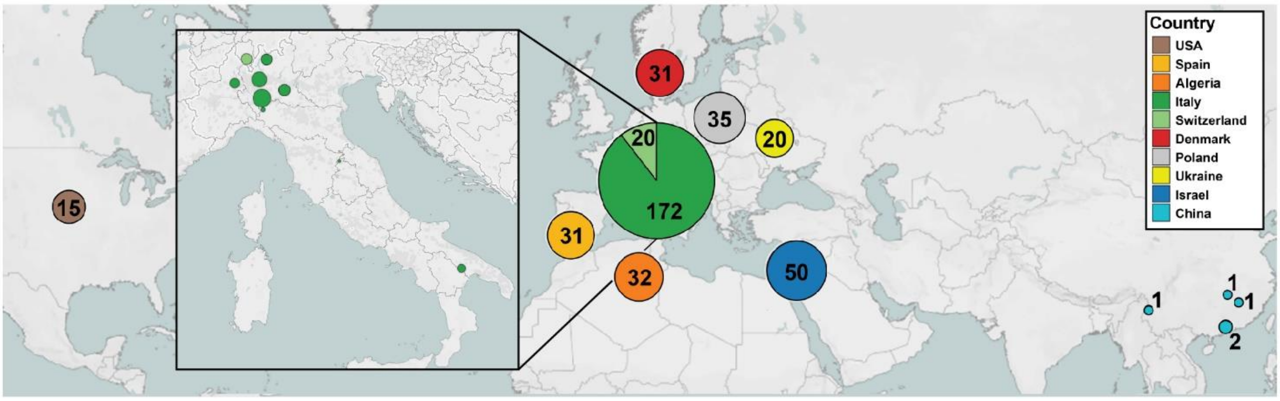

Fig. S3. Nucleotide diversity in barn swallow mitogenome loci. The linearized gene map of the barn swallow mitogenome is shown above. Protein-coding genes are in green, rRNA genes in light blue, tRNA genes in blue and the two control regions in red. Nucleotide diversity (π) per position (continuous red line) calculated with DnaSP v6 on the entire mitogenome (100-bp windows, step size of 25 sites) (Rozas et al. 2017) is shown below.

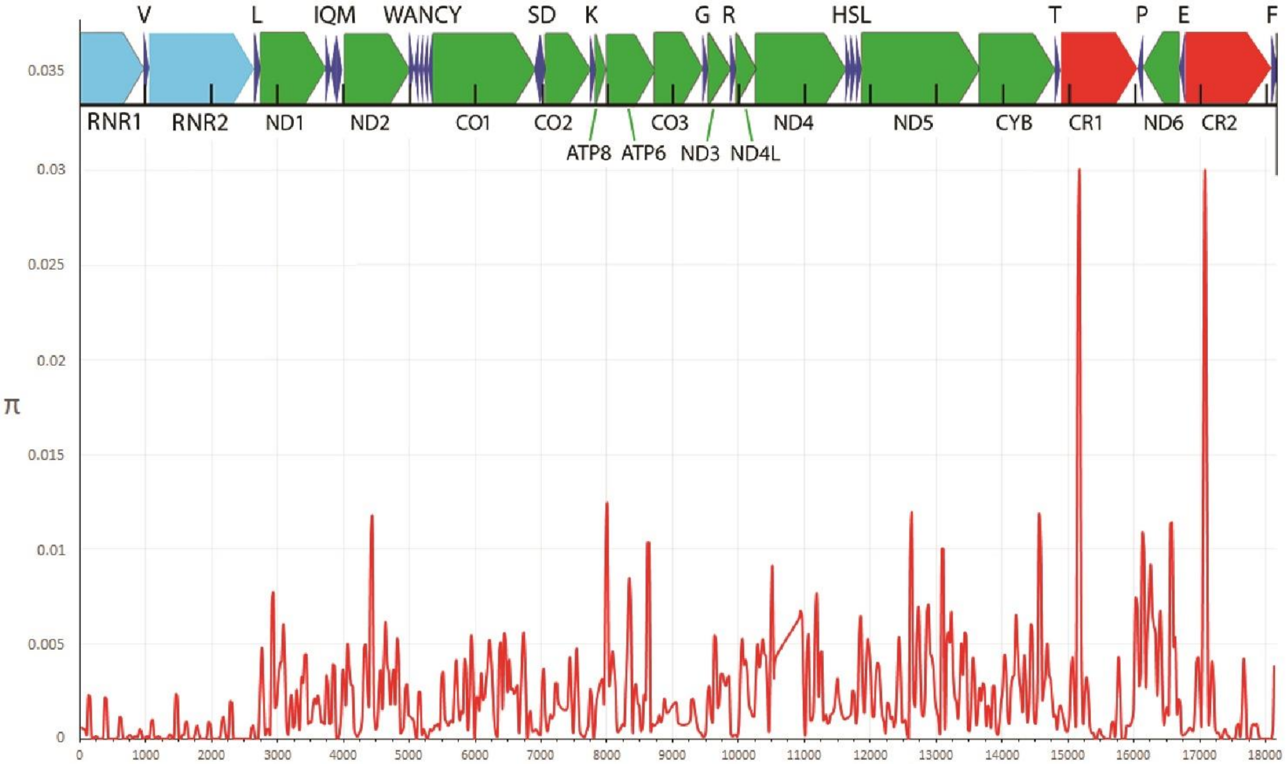

Fig. S4. The ratio of divergence at non-synonymous and synonymous sites (dN/dS) in mtDNA protein-coding genes. This chart was constructed using mtDNA GeneSyn v1.0 (Pereira et al. 2009). Differences in terms of dN/dS ratio are indicative of purifying selection. We compared these values on the entire tree (grey bars) and by comparing branches that differentiated before and after the end (~11.7 kya) of the Younger Dryas; red (pre-YD) and blue (post-YD) bars, respectively.

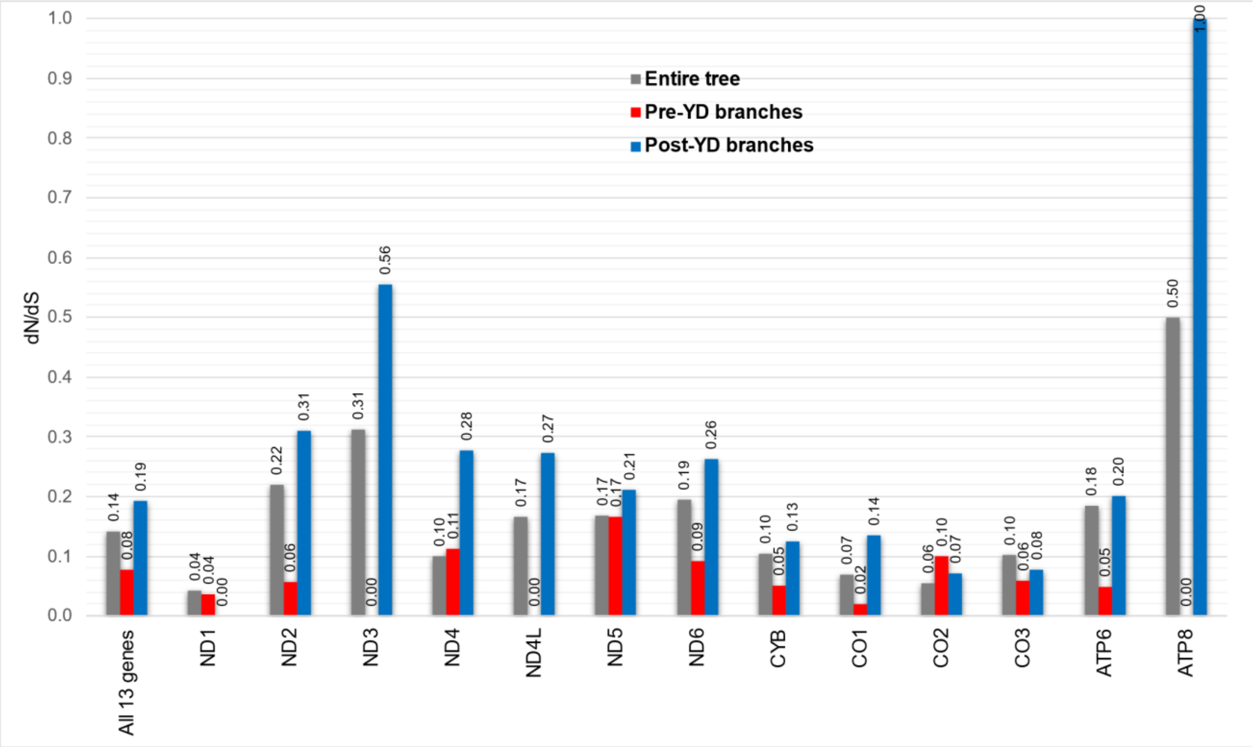

Fig. S5. Detailed maximum parsimony phylogeny of *Hirundo rustica* mitogenomes. This tree was built using the entire mitogenome coding-region of the 411 barn swallow samples and was rooted using *H. angolensis* and *H. aethiopica* reference mitogenomes (NC_050287 and NC_050293). Main haplogroup and sub-haplogroup affiliations are shown. With the exception of A3, sub-haplogroups were named only when encompassing at least two haplotypes. Subspecies affiliations are according to the colours in the legend. Mutations, relative to HrrRS, are transitions unless a base is explicitly indicated. Suffixes indicate transversions (to A, G, C, or T). Reversions are marked with "@" and recurring mutations are underlined. Both sample ID names (in green) and IDs employed in phylogenetic analyses are provided. Heteroplasmic positions are shown below sample names.

EXCEL FILE

Fig. S6. Phylogenetic distribution of 31 barn swallow mitogenomes from Denmark. This tree is identical to the one in fig. 2, but the mitogenomes marked in black are from Denmark. The three sub-haplogroups A1a1a1a, A1a2e1a1a5 and A1a2f1b are over-represented in the Danish specimens compared to the other European populations.

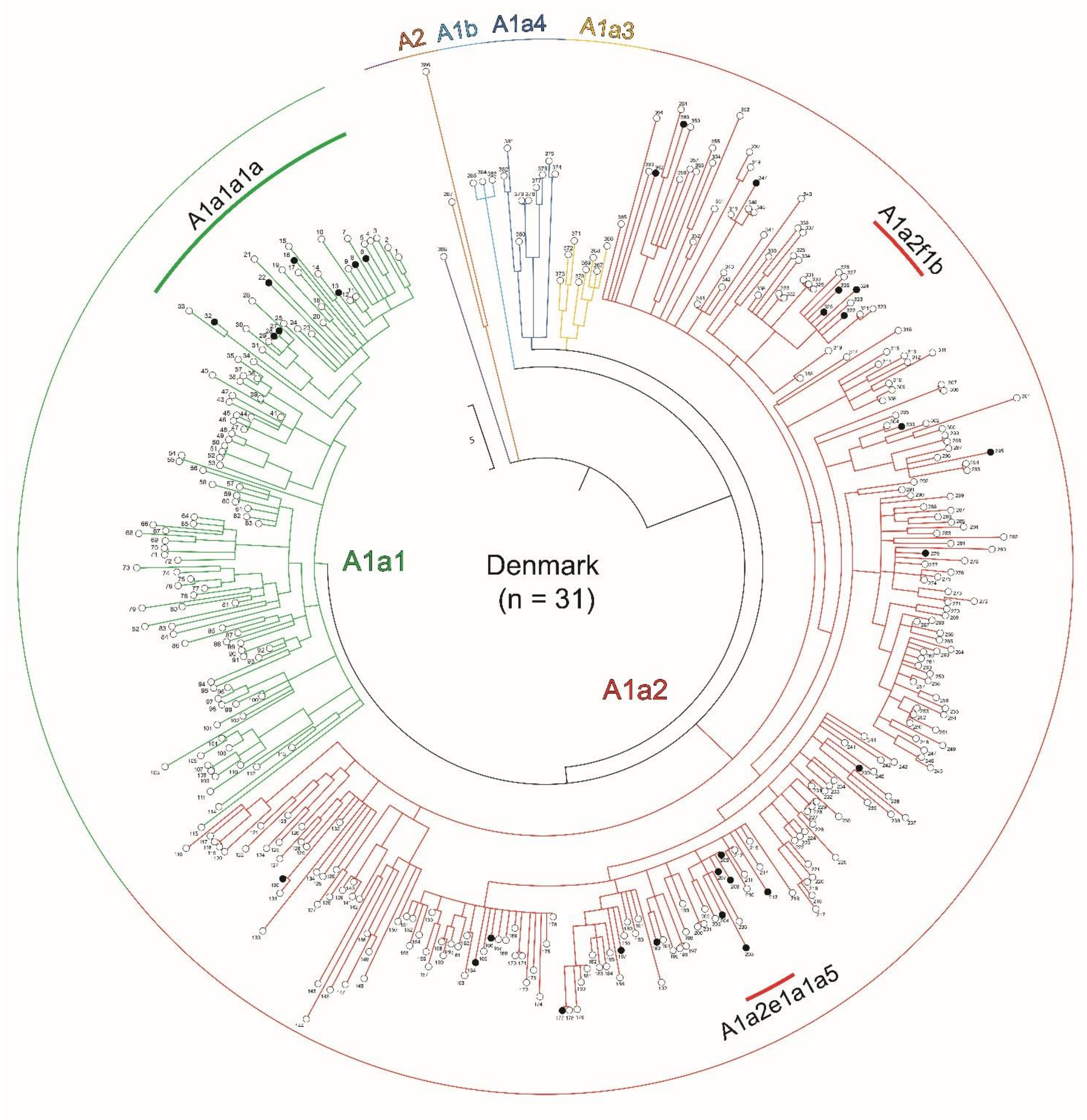

Fig. S7. Correlation between mitogenome diversity and latitude. Haplotype diversity (HD) and nucleotide diversity (Pi) are shown in *H. r. rustica* and *H. r. transitiva* populations for haplogroup A1 and its major sub-haplogroups A1a1 and A1a2. Populations are coded as follows: DK, Denmark; DZ, Algeria, CH, Switzerland; ES, Spain; IL, Israel; N_IT, North Italy; PL, Poland; UA, Ukraine; S_IT, South Italy. The grayscale within the plots on the left indicates the geographical macro-groups (North, Center and South) in which populations were clustered (plots on the right) to reduce possible bias due to size differences of population samples. HD was extremely high in almost all populations, with a slightly lower value in Israel (*H. r. transitiva*) due to some haplotype sharing in samples from the same location, thus possibly maternally related. Correlations between HD and latitude were not significant. Negative (not significant) correlations were instead detected when Pi was considered, especially when it was calculated considering only unique haplotypes. A significant negative correlation (p-value < 0.05) close to 1 for haplogroup A1 was observed when assessing geographical macro-groups.

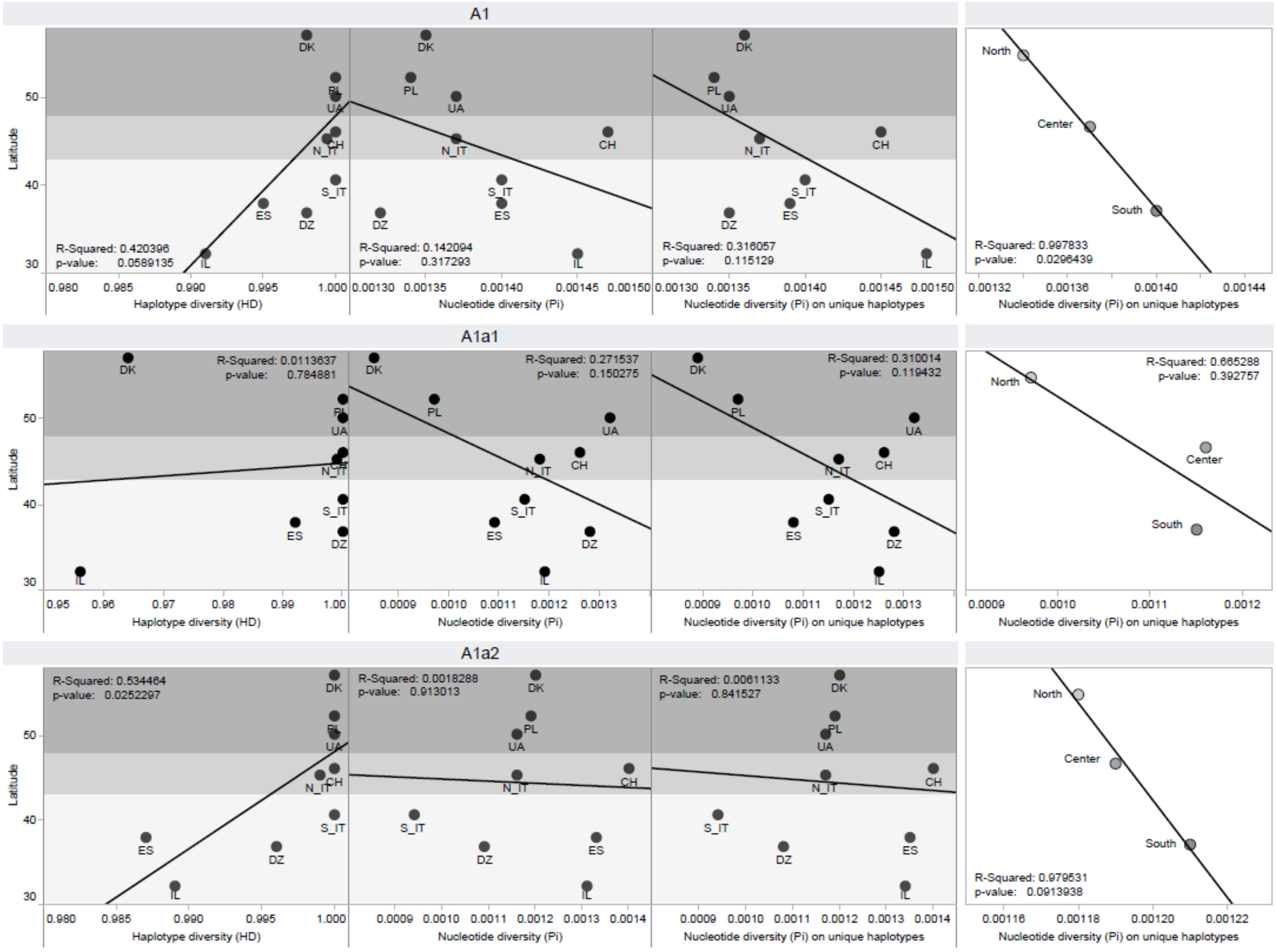

Fig. S8. Schematic maximum parsimony phylogeny of *Hirundo* mitogenomes. This tree was built as in fig. 1 using the same samples plus additional published *Hirundo* mitogenomes (Carter et al. 2020). It was rooted with *Progne chalybea* (NC_020605) and *Delichon urbicum* (NC_050298) mitogenomes. HrAM refers to the *Hirundo rustica* Ancestral Mitogenome.

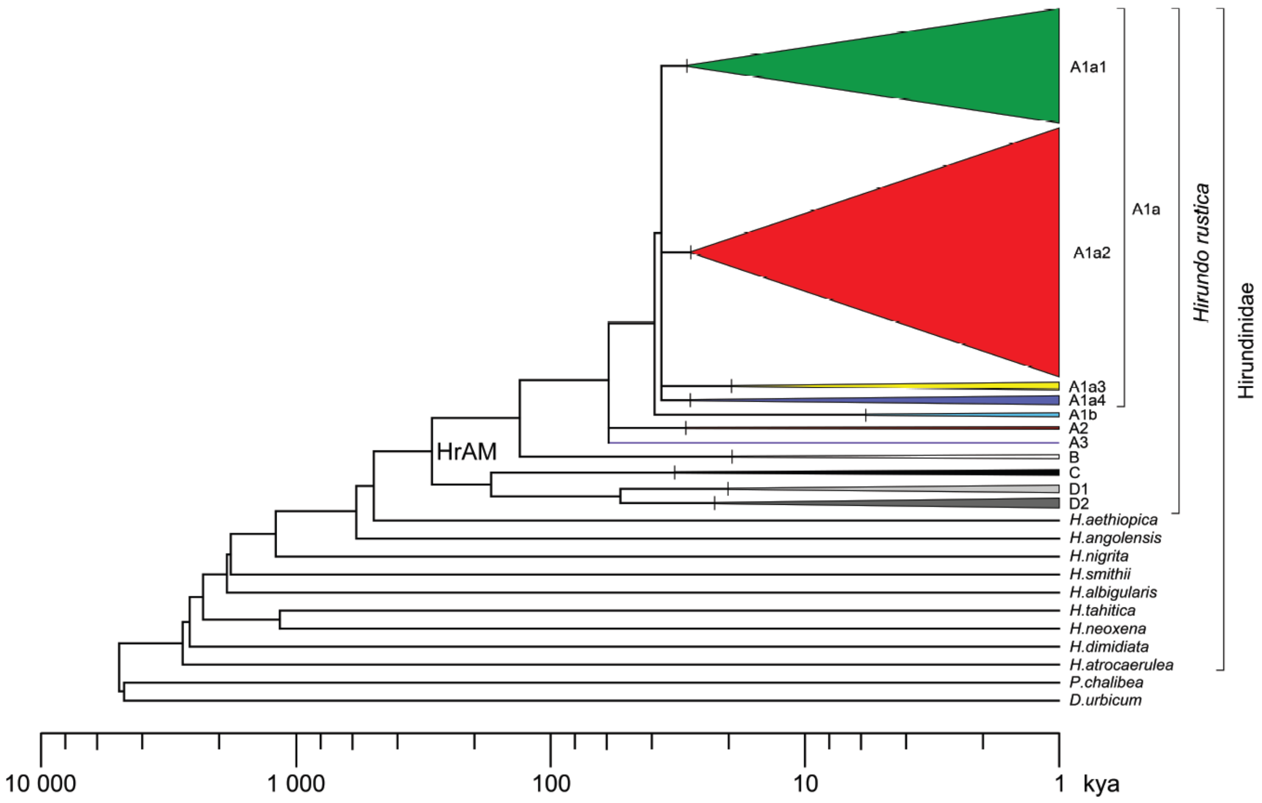

Fig. S9. Mantel correlation tests between genetic and geographic distances. Histograms represent permuted values (under the absence of spatial structure) for haplogroups A1a1 (A), A1a2 (B) and A1 (C). The original value of the correlation between the distance matrices is represented by the black rhombus. Isolation by distance (IBD) is present when the original value is out of the reference distribution. Scatterplots with point densities represent the correlation between the two distances for haplogroups A1a1 (D), A1a2 (E) and A1 (F). The regression line is reported in blue and its 95% confidence interval in grey. The Mantel *r* values with the corresponding *p-value* and the linear regression r^2 values are reported for each plot.

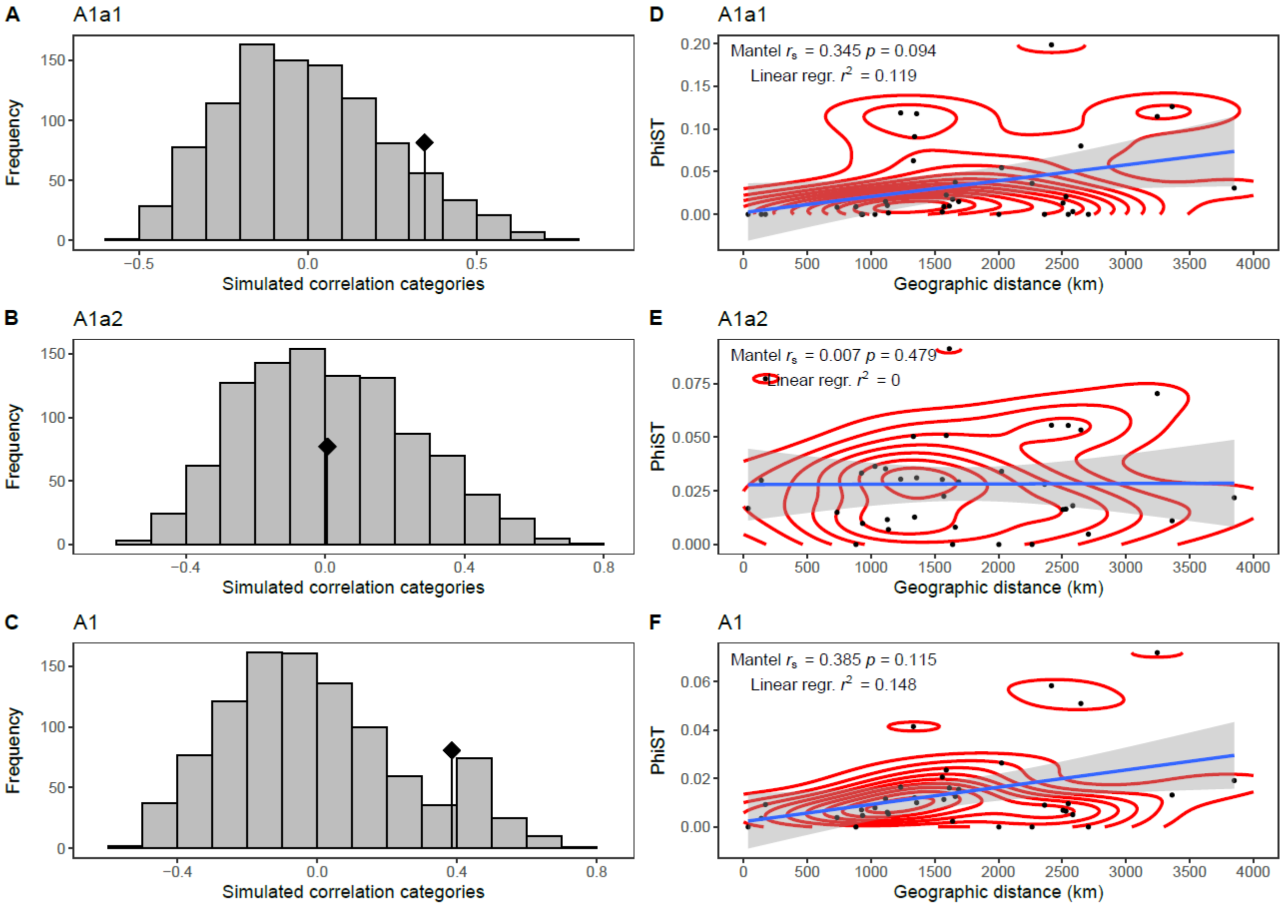

SUPPLEMENTARY TABLES

Supplementary Table 1. Barn swallow samples analysed for mtDNA variation.

EXCEL FILE

Supplementary Table 2. Locus map of the barn swallow mitogenome.

Map Locus	Strand	Starting np [a]	Ending np [a]	Length (bp)	Description
MT-RNR1	+	1	972	972	12S ribosomal RNA
MT-TV	+	973	1042	70	tRNA valine
MT-RNR2	+	1043	2650	1608	16S ribosomal RNA
MT-TL1	+	2651	2725	75	tRNA leucine1
MT-ND1	+	2753	3730	978	NADH dehydrogenase subunit 1
MT-TI	+	3738	3808	71	tRNA isoleucine
MT-TQ	-	3816	3887	72	tRNA glutamine
MT-TM	+	3887	3955	69	tRNA methionine
MT-ND2	+	3956	4996	1041	NADH dehydrogenase subunit 2
MT-TW	+	4997	5066	70	tRNA tryptophan
MT-TA	-	5068	5136	69	tRNA alanine
MT-TN	-	5146	5218	73	tRNA asparagine
MT-TC	-	5219	5284	66	tRNA cysteine
MT-TY	-	5284	5354	71	tRNA tyrosine
MT-CO1	+	5356	6912	1557	Cytochrome c oxidase subunit I
MT-TS1	-	6904	6978	75	tRNA serine1
MT-TD	+	6983	7051	69	tRNA aspartic acid
MT-CO2	+	7062	7745	684	Cytochrome c oxidase subunit II
MT-TK	+	7747	7817	71	tRNA lysine
MT-ATP8	+	7819	7986	168	ATP synthase subunit 8
MT-ATP6	+	7977	8660	684	ATP synthase subunit 6
MT-CO3	+	8667	9450	784	Cytochrome c oxidase subunit III
MT-TG	+	9451	9519	69	tRNA glycine
MT-ND3	+	9520	9870	351	NADH dehydrogenase subunit 3
MT-TR	+	9873	9942	70	tRNA arginine
MT-ND4L	+	9944	10240	297	NADH dehydrogenase subunit 4L
MT-ND4	+	10234	11611	1378	NADH dehydrogenase subunit 4
MT-TH	+	11612	11680	69	tRNA histidine

MT-TS2	+	11681	11747	67	tRNA serine2
MT-TL2	+	11747	11817	71	tRNA leucine2
MT-ND5	+	11818	13635	1818	NADH dehydrogenase subunit 5
MT-CYB	+	13644	14786	1143	Cytochrome b
MT-TT	+	14791	14859	69	tRNA threonine
MT-CR1	+	14860	16067	1208	control region 1
MT-TP	-	16068	16137	70	tRNA proline
MT-ND6	-	16149	16667	519	NADH dehydrogenase subunit 6
MT-TE	-	16669	16740	72	tRNA glutamic acid
MT-CR2	+	16741	18074	1334	control region 2
MT-TF	+	18075	18143	69	tRNA phenylalanine

[a] Nucleotide positions obtained from mitogenome #20, *H. r. rustica* reference sequence (HrrRS, MZ905359).

Supplementary Table 3. ML and Bayesian age estimates for barn swallow haplogroups and sub-haplogroups. Estimates are based on the MP phylogeny (411 mtDNAs, only coding regions, 15601 bps) of supplementary fig. S5, Supplementary Material online. ML estimates were obtained by subdividing the mitogenome coding region into 17 partitions (13 for protein-coding genes, one for all tRNAs, one for each rRNA gene, and one for all intergenic regions). In the Bayesian estimates, substitution rate values obtained with ML were employed without node age constraints.

Haplogroups /Sub-haplogroups	Number of mtDNAs	ML		Bayesian	
		T (ky)	ΔT (ky)	T (ky)	ΔT (ky)
ABCD	411	291.4	12.1	276.9	24.3
>AB	392	115.9	5.2	115.6	13.3
>>A	388	56.3	2.6	57.1	6.4
>>>A1	385	34.7	1.6	38.0	3.8
>>>>A1a	382	31.3	1.4	34.3	2.9
>>>>>A1a1	115	27.6	1.2	28.9	2.4
>>>>>>A1a1a	55	23.9	1.2	24.5	2.0
>>>>>>>A1a1a1	34	21.4	1.2	21.4	2.1
>>>>>>>>A1a1a1a	33	19.3	1.1	19.5	2.0
>>>>>>>>>A1a1a1a1	25	16.3	0.8	17.2	1.8
>>>>>>>>>>A1a1a1a1a	13	12.2	0.7	13.1	1.5
>>>>>>>>>>>A1a1a1a1a1	3	9.0	0.7	9.1	1.7
>>>>>>>>>>>>A1a1a1a1a1a	2	5.0	0.6	5.2	2.2
>>>>>>>>>>>A1a1a1a1a2	6	9.7	0.6	10.7	1.2
>>>>>>>>>>>>A1a1a1a1a2a	2	7.1	0.6	6.9	2.0
>>>>>>>>>>>A1a1a1a1a3	3	9.4	0.8	9.0	1.7
>>>>>>>>>>A1a1a1a1b	2	8.1	0.7	8.0	2.2
>>>>>>>>>>A1a1a1a1c	2	14.3	0.8	11.1	2.1
>>>>>>>>>>A1a1a1a1d	2	8.1	0.8	7.7	2.3
>>>>>>>>>A1a1a1a2	8	15.6	1.0	15.7	2.1
>>>>>>>>>>A1a1a1a2a	6	8.3	0.7	11.2	1.7
>>>>>>>>>>>A1a1a1a2a1	5	5.8	0.6	9.1	1.6
>>>>>>>>>>A1a1a1a2b	2	4.1	0.5	5.0	2.3

>>>>>>>A1a1a2	13	12.0	0.8	15.4	2.2
>>>>>>>>A1a1a2a	4	5.0	0.6	9.2	2.0
>>>>>>>>>A1a1a2a1	3	2.7	0.4	6.7	2.2
>>>>>>>>A1a1a2b	6	8.1	0.6	11.1	1.6
>>>>>>>>>A1a1a2b1	2	4.4	0.5	5.3	2.1
>>>>>>>>A1a1a2c	3	1.9	0.3	5.6	2.6
>>>>>>>A1a1a3	6	15.8	1.2	14.6	2.8
>>>>>>>>A1a1a3a	5	8.9	1.0	9.5	2.0
>>>>>>>>>A1a1a3a1	2	2.8	0.4	3.5	1.9
>>>>>>A1a1b	40	21.6	1.0	22.6	2.2
>>>>>>>A1a1b1	29	19.6	0.9	20.1	2.1
>>>>>>>>A1a1b1a	8	9.0	0.6	12.9	1.9
>>>>>>>>>A1a1b1a1	3	7.6	0.6	9.7	1.5
>>>>>>>>A1a1b1b	9	12.0	0.7	13.2	1.8
>>>>>>>>>A1a1b1b1	4	9.0	0.6	10.0	1.3
>>>>>>>>>A1a1b1b2	2	6.5	0.6	7.0	2.2
>>>>>>>>A1a1b1c	12	18.2	0.9	17.2	2.0
>>>>>>>>>A1a1b1c1	9	15.6	0.8	14.3	1.8
>>>>>>>>>>A1a1b1c1a	5	11.0	0.7	10.8	1.4
>>>>>>>>>>>A1a1b1c1a1	2	9.4	0.7	8.8	1.5
>>>>>>>>>>>A1a1b1c1a2	3	6.2	0.7	7.7	2.0
>>>>>>>>>>>>A1a1b1c1a2a	2	1.2	0.4	2.9	1.6
>>>>>>>>>>A1a1b1c1b	2	13.6	0.8	10.8	1.8
>>>>>>>>>>A1a1b1c1c	2	10.6	1.0	9.9	1.7
>>>>>>>>>A1a1b1c2	2	16.6	0.9	13.4	2.4
>>>>>>>A1a1b2	5	15.8	0.9	14.6	2.4
>>>>>>>>A1a1b2a	3	12.7	0.8	11.1	1.8
>>>>>>>>>A1a1b2a1	2	10.6	0.8	9.1	1.6
>>>>>>>A1a1b3	6	16.5	1.2	14.7	2.6
>>>>>>>>A1a1b3a	4	7.1	0.9	9.8	1.9

>>>>>>>>A1a1b3b	2	14.3	1.0	11.4	2.2
>>>>>>A1a1c	8	15.4	0.9	15.2	2.7
>>>>>>>A1a1c1	3	5.1	0.7	6.0	2.4
>>>>>>>>A1a1c1a	2	1.6	0.4	2.2	1.5
>>>>>>>A1a1c2	2	3.7	0.6	5.5	2.5
>>>>>>A1a1d	8	18.9	1.1	18.4	2.9
>>>>>>>A1a1d1	7	8.0	0.6	10.8	1.7
>>>>>>>>A1a1d1a	3	4.6	0.5	6.3	2.2
>>>>>>>>>A1a1d1a1	2	2.5	0.4	3.1	1.7
>>>>>>>>A1a1d1b	3	4.2	0.6	5.9	2.1
>>>>>>A1a1e	3	21.8	1.2	18.5	3.3
>>>>>>>A1a1e1	2	12.0	1.2	10.4	2.4
>>>>>A1a2	250	26.7	1.1	29.4	2.1
>>>>>>A1a2a	5	11.9	0.8	12.4	2.1
>>>>>>>A1a2a1	4	9.4	0.8	9.9	1.5
>>>>>>A1a2b	6	20.7	1.2	17.8	2.9
>>>>>>>A1a2b1	5	17.5	1.0	14.7	2.4
>>>>>>>>A1a2b1a	3	14.2	0.9	11.6	2.0
>>>>>>>>>A1a2b1a1	2	9.7	0.8	8.3	1.9
>>>>>>>>A1a2b1b	2	9.4	0.9	8.8	2.0
>>>>>>>A1a2c	10	18.1	0.9	17.1	2.7
>>>>>>>>A1a2c1	3	14.2	1.0	10.8	2.1
>>>>>>>>>A1a2c1a	2	8.3	0.8	7.0	2.2
>>>>>>>>A1a2c2	2	1.2	0.3	1.8	1.3
>>>>>>>A1a2d	14	25.0	1.1	23.0	2.4
>>>>>>>>A1a2d1	2	7.1	0.7	8.0	2.5
>>>>>>>>A1a2d2	4	12.4	1.0	11.4	2.5
>>>>>>>>>A1a2d2a	3	1.9	0.3	3.7	2.0
>>>>>>>>A1a2d3	8	20.9	1.0	17.8	2.5
>>>>>>>>>A1a2d3a	4	19.6	0.9	14.9	2.4

>>>>>>>>>>A1a2d3a1	2	18.4	0.9	12.6	2.3
>>>>>>>>>A1a2d3b	2	6.4	0.7	6.4	2.5
>>>>>>>A1a2e	167	24.1	1.1	25.4	1.9
>>>>>>>>A1a2e1	157	21.4	1.0	22.3	1.7
>>>>>>>>>A1a2e1a	142	17.0	0.8	20.6	1.6
>>>>>>>>>>A1a2e1a1	94	13.5	0.6	18.3	1.5
>>>>>>>>>>>A1a2e1a1a	66	11.5	0.5	16.2	1.3
>>>>>>>>>>>>A1a2e1a1a1	26	7.8	0.4	12.5	1.2
>>>>>>>>>>>>>A1a2e1a1a1a	4	4.8	0.4	7.7	2.0
>>>>>>>>>>>>>A1a2e1a1a1b	3	5.5	0.5	7.2	2.1
>>>>>>>>>>>>>>A1a2e1a1a1b1	2	4.2	0.4	5.0	1.9
>>>>>>>>>>>>>A1a2e1a1a1c	4	5.5	0.5	7.4	2.0
>>>>>>>>>>>>>>A1a2e1a1a1c1	3	3.2	0.4	4.3	1.8
>>>>>>>>>>>>>>>A1a2e1a1a1c1a	2	1.2	0.2	1.7	1.2
>>>>>>>>>>>>>A1a2e1a1a1d	2	3.5	0.4	4.4	2.0
>>>>>>>>>>>>A1a2e1a1a2	16	10.1	0.5	12.8	1.4
>>>>>>>>>>>>>A1a2e1a1a2a	12	8.7	0.5	11.0	1.2
>>>>>>>>>>>>>>A1a2e1a1a2a1	9	7.3	0.4	9.6	1.1
>>>>>>>>>>>>>>>A1a2e1a1a2a1a	5	3.5	0.4	5.6	1.6
>>>>>>>>>>>>>>>A1a2e1a1a2a1b	3	4.6	0.4	6.0	2.0
>>>>>>>>>>>>>A1a2e1a1a3	5	8.1	0.6	10.2	1.4
>>>>>>>>>>>>>>A1a2e1a1a3a	2	5.5	0.6	6.2	2.2
>>>>>>>>>>>>>A1a2e1a1a4	4	8.5	0.7	10.0	1.5
>>>>>>>>>>>>>A1a2e1a1a5	8	8.5	0.5	11.5	1.4
>>>>>>>>>>>>>>A1a2e1a1a5a	2	6.2	0.5	7.0	2.2
>>>>>>>>>>>>>>A1a2e1a1a5b	3	7.4	0.5	9.4	1.3
>>>>>>>>>>>>>A1a2e1a1a6	3	5.5	0.6	7.9	2.3
>>>>>>>>>>>A1a2e1a1b	14	11.0	0.6	13.2	1.8
>>>>>>>>>>>>A1a2e1a1b1	5	6.5	0.5	8.8	1.7
>>>>>>>>>>>>>A1a2e1a1b1a	3	2.1	0.3	3.4	1.5

>>>>>>>>>>>>A1a2e1a1b2	3	3.0	0.4	4.8	2.2
>>>>>>>>>>>>A1a2e1a1b3	2	6.4	0.6	6.7	2.3
>>>>>>>>>>>A1a2e1a1c	2	1.8	0.4	2.8	2.0
>>>>>>>>>>>A1a2e1a1d	3	9.7	0.7	10.0	1.7
>>>>>>>>>>>A1a2e1a1e	5	11.9	0.6	12.8	1.9
>>>>>>>>>>>>A1a2e1a1e1	3	8.0	0.5	9.1	1.6
>>>>>>>>>>A1a2e1a2	46	12.9	0.6	16.7	1.6
>>>>>>>>>>>A1a2e1a2a	26	10.6	0.6	13.9	1.5
>>>>>>>>>>>>A1a2e1a2a1	24	8.5	0.5	12.4	1.3
>>>>>>>>>>>>>A1a2e1a2a1a	20	6.7	0.4	11.1	1.1
>>>>>>>>>>>>>>A1a2e1a2a1a1	3	4.1	0.4	5.8	1.9
>>>>>>>>>>>>>>>A1a2e1a2a1a1a	2	1.1	0.2	1.7	1.2
>>>>>>>>>>>>>>A1a2e1a2a1a2	2	5.3	0.4	7.1	1.9
>>>>>>>>>>>>>>A1a2e1a2a1a3	5	4.8	0.4	7.4	1.8
>>>>>>>>>>>>>>>A1a2e1a2a1a3a	4	1.4	0.2	4.3	1.7
>>>>>>>>>>>>>>A1a2e1a2a1a4	2	3.2	0.4	4.2	1.9
>>>>>>>>>>>>>>>A1a2e1a2a1a5	2	1.8	0.4	2.7	1.8
>>>>>>>>>>>>>A1a2e1a2a1b	3	4.1	0.5	6.9	2.4
>>>>>>>>>>>>A1a2e1a2a2	2	5.3	0.6	7.6	2.5
>>>>>>>>>>>A1a2e1a2b	10	10.4	0.6	12.0	1.6
>>>>>>>>>>>>A1a2e1a2b1	3	6.2	0.6	6.9	2.1
>>>>>>>>>>>>>A1a2e1a2b1a	2	2.3	0.4	2.7	1.5
>>>>>>>>>>>>A1a2e1a2b2	3	3.0	0.4	4.5	2.0
>>>>>>>>>>>A1a2e1a2c	2	11.5	0.6	10.9	2.0
>>>>>>>>>>>A1a2e1a2d	2	7.8	0.7	8.3	2.2
>>>>>>>>>>>A1a2e1a2e	4	9.4	0.7	10.4	1.5
>>>>>>>>>>>>A1a2e1a2e1	2	6.7	0.7	6.8	2.1
>>>>>>>>>>A1a2e1a3	2	12.2	1.0	11.6	2.8
>>>>>>>>>A1a2e1b	12	17.5	0.9	18.0	2.0
>>>>>>>>>>A1a2e1b1	7	11.3	0.8	12.4	1.7

>>>>>>>>>>>A1a2e1b1a	5	9.4	0.7	10.3	1.3
>>>>>>>>>>A1a2e1b2	5	15.0	0.9	14.5	2.1
>>>>>>>>>>>A1a2e1b2a	3	11.9	0.8	11.1	1.8
>>>>>>>>>>>>A1a2e1b2a1	2	9.0	0.7	8.5	1.7
>>>>>>>>>>>A1a2e1b2b	2	1.9	0.5	3.5	2.6
>>>>>>>>A1a2e2	8	10.8	0.7	13.0	2.0
>>>>>>>>>A1a2e2a	2	1.8	0.4	2.7	1.9
>>>>>>>>>A1a2e2b	2	6.9	0.6	7.4	2.2
>>>>>>>>>A1a2e2c	2	6.9	0.6	7.4	2.2
>>>>>>>>A1a2e3	2	21.6	1.1	19.5	3.3
>>>>>>>A1a2f	23	23.9	1.1	22.2	2.5
>>>>>>>>A1a2f1	19	11.0	0.6	14.7	1.9
>>>>>>>>>A1a2f1a	2	4.4	0.6	4.9	2.3
>>>>>>>>>A1a2f1b	9	7.6	0.4	11.1	1.3
>>>>>>>>>>A1a2f1b1	2	1.2	0.4	3.8	1.7
>>>>>>>>A1a2f2	3	15.6	1.1	13.8	2.6
>>>>>>>>>A1a2f2a	2	9.4	0.8	8.8	2.0
>>>>>>>A1a2g	4	9.7	0.8	11.6	2.3
>>>>>>>A1a2h	4	3.5	0.5	6.7	2.7
>>>>>>>A1a2i	2	3.4	0.5	4.4	2.4
>>>>>>>A1a2j	2	7.8	0.8	8.5	2.5
>>>>>>>A1a2k	3	25.3	1.1	22.7	2.9
>>>>>>>>A1a2k1	2	14.9	0.9	12.3	2.6
>>>>>>>A1a2l	6	20.0	1.0	18.2	2.6
>>>>>>>>A1a2l1	4	17.3	0.9	14.2	2.4
>>>>>>>>A1a2l2	2	9.6	0.7	9.3	1.9
>>>>>A1a3	8	18.4	1.3	19.5	3.7
>>>>>>A1a3a	5	13.3	1.1	14.0	2.9
>>>>>>>A1a3a1	4	7.4	0.7	9.5	2.1
>>>>>>>>A1a3a1a	3	5.1	0.5	6.9	2.2

>>>>>>A1a3b	3	10.6	0.9	11.5	2.5
>>>>>>>A1a3b1	2	6.2	0.6	6.9	2.4
>>>>>A1a4	9	28.9	1.3	26.1	3.3
>>>>>>A1a4a	2	1.2	0.4	2.2	1.6
>>>>>>A1a4b	4	12.9	1.2	11.8	2.6
>>>>>>>A1a4b1	2	3.7	0.5	4.1	2.1
>>>>>>A1a4c	3	14.5	1.1	12.5	2.9
>>>>>>>A1a4c1	2	4.2	0.6	4.3	2.1
>>>>A1b	3	2.7	0.4	5.7	2.8
>>>A2	2	34.0	1.9	29.5	5.5
>>>A3	1	-	-	-	-
>>B	4	19.5	1.2	18.9	3.9
>>>B1	2	11.5	0.9	10.6	2.3
>CD	19	165.5	7.1	156.4	18.0
>>C	4	38.6	2.1	31.1	5.7
>>>C1	3	28.5	1.7	22.0	4.1
>>>>C1a	2	18.2	1.3	12.6	3.0
>>D	15	54.0	2.6	51.1	7.9
>>>D1	6	20.0	1.3	19.7	3.9
>>>>D1a	4	16.3	1.0	14.8	3.0
>>>>>D1a1	2	9.7	0.8	8.7	2.1
>>>>D1b	2	2.5	0.4	3.4	2.2
>>>D2	9	20.2	1.0	20.6	3.4
>>>>D2a	3	8.3	0.7	10.5	2.0
>>>>D2b	2	13.1	0.8	9.6	2.0
>>>>D2c	3	21.2	1.0	12.5	2.7

Supplementary Table 4. Nucleotide diversity (%) within and between barn swallows belonging to different haplogroups and from different geographic areas. Intragroup nucleotide diversities (π) are on the diagonal.

Haplogroup / Source	A			B	C	D
	Europe / N. Africa N = 341	Israel N = 46	Total [a] N = 388	Israel N = 4	China N = 4	USA N = 15
A Europe/N. Africa (N = 341)	0.13 ± 0.002	0.13 ± 0.004	0.13 ± 0.002	0.53 ± 0.02	1.21 ± 0.045	1.19 ± 0.025
A Israel (N = 46)	-	0.13 ± 0.008	0.13 ± 0.004	0.53 ± 0.057	1.21 ± 0.129	1.19 ± 0.070
A Total [a] (N = 388)	-	-	0.13 ± 0.002	0.53 ± 0.019	1.21 ± 0.043	1.19 ± 0.023
B Israel (N = 4)	-	-	-	0.09 ± 0.017	1.19 ± 0.365	1.18 ± 0.205
C China (N = 4)	-	-	-	-	0.14 ± 0.028	0.70 ± 0.121
D USA (N = 15)	-	-	-	-	-	0.17 ± 0.013

[a] It includes also the single haplogroup A mitogenome from China (# 258).

Supplementary Table 5. Oligonucleotides employed for PCR amplification of barn swallow mitogenomes in eleven fragments.

PCR fragment	Primer name	5'np [a]	3'np [a]	Sequence 5'→ 3'	T_m (ºC)
1	49FOR	49	68	GCAAGTATCCGCATTCCAGT	60.1
	1868REV	1868	1849	ACGGGTTTGCCTAGTTCCTT	60.0
2	1581FOR	1581	1600	CAAGCAGCCATCAACAAAGA	60.0
	3463REV	3463	3444	GTTGGCGTATTCGGCTAAAA	60.1
3	3180FOR	3180	3199	TTGCCCAGACCATCTCGTAT	60.5
	5078REV	5078	5059	TGCGGGTCTTAGCAGAAACT	60.0
4	4721FOR	4721	4740	CCCCTAACAGGCTTCCTACC	60.0
	6554REV	6554	6535	AGGGGGAATCAGTGTGTGAA	60.4
5	6287FOR	6287	6306	TAATCATTGCCATCCCCACT	60.2
	8185REV	8185	8166	GCCCATTTGTGTCCTTTGTT	59.8
6	7803FOR	7803	7822	TCCCCTCCTTAATGACATGC	59.9
	9612REV	9612	9593	GTCTGGGGTTATTTGGGCTA	58.9
7	9244FOR	9244	9263	ACGGATCCACATTCTTCGTC	59.9
	11080REV	11080	11061	TTAGGTCGATTTGGCGTAGG	60.1
8	10770FOR	10770	10789	CAAGCTCTCACACCCAACAA	59.9
	12585REV	12585	12566	GCGACAACTATTGTGCTGGA	59.9
9	12304FOR	12304	12323	CGAGCAGAAGCCAATACCTC	60.0
	14179REV	14179	14160	ATCGGGTTAGGGTAGGGTTG	60.0
10	13901FOR	13901	13920	ACGGAGCCTCCTTCTTCTTC	60.0
	15624REV	15624	15605	CACCTGCTACGCACTTTGAA	60.1
11	15271FOR	15271	15290	TAAAGCCAACTGCTCGTCCT	60.0
	774REV	774	755	CCCCTTTGCCGTATGTTCTA	60.0

[a] Nucleotide positions obtained from mitogenome #20, *H. r. rustica* reference sequence (HrrRS, MZ905359).

Supplementary Table 6. Oligonucleotides employed for the Sanger sequencing of the first five barn swallow mitogenomes.

PCR fragment [a]	Primer name	5'np [b]	3'np [b]	Sequence 5'→ 3'	T_m (ºC)
1	112FOR	112	131	TCAGGCACACCATAACTGTA	55.1
	592FOR	592	611	CCACGATACACCTAACCATT	55.0
	1066FOR	1066	1085	CTGACCTGGAATAACAAAGC	55.0
2	1703FOR	1703	1722	TAACCTGGGTACTTCCCTCT	55.3
	2114FOR	2114	2133	AGAAGACCCTGTGGAACTTT	55.4
	2629FOR	2629	2648	CCCAAATCCTAGAAAAGGAC	55.4
3	3236FOR	3236	3255	CTACTCAGCGGAAGCTACAC	55.4
	3738FOR	3738	3757	GGAAATGTGCCTGAACTTAG	55.0
	4324FOR	4324	4343	TATCATCACCGGTCTTATCC	54.9
4	4807FOR	4807	4826	ACTATCCCTACTCGGCCTAT	54.6
	5344FOR	5344	5363	GCCATCTTACCTGTGACATT	551
	5894FOR	5894	5913	ACCAAACACCACTATTCGTC	55.0
5	6344FOR	6344	6363	GCGGAATTATCAAATGAGAC	54.8
	6969FOR	6969	6988	CTTCTTTCTCACAAGGGATG	54.9
	7525FOR	7525	7544	CTAACGACGTACTGCACTCA	55.1
6	7870FOR	7870	7889	CTGACATTCTCCCTCATCAT	54.9
	8249FOR	8249	8268	TACTTTTACCCCAACCACAC	55.0
	8778FOR	8778	8797	AACACCCCATACCTCCTAAT	55.0
7	9293FOR	9293	9312	CATCGGATCAACATTCCTAC	53.6
	9840FOR	9840	9859	GGCTCAAGGAGGACTAGAAT	55.1
	10399FOR	10399	10418	ACATCAATCGACCAAATCTC	54.9
8	10825FOR	10825	10844	GCCCTTTTAGTAGCCTTCAT	55.3
	11557FOR	11557	11576	ATCCCAATAGTCCTCCTCAT	55.0
	12050FOR	12050	12069	TCCCAATCAGCCTAAAAATA	55.0
9	12358FOR	12358	12377	GACATCGGACTCATTCTCTG	55.6
	12866FOR	12866	12885	AACCTCAATGGAGAACAAGA	54.7

	13416FOR	13416	13435	TAACACATCGATCCAGCATA	55.1
10	13941FOR	13941	13960	CGGACGAGGATTCTACTATG	54.9
	14492FOR	14492	14511	GATCCATCCCAAACAAACTA	55.0
	15516REV	15516	15497	CAGCAGCTGTATCTGTGAAG	54.6
11	15367FOR	15367	15386	ACGTCAGTTATGCTTTCGTT	55.1
	15775FOR	15775	15794	GCACTGGATAATGAAATGGT	55.0
	424REV	424	405	GCTTTCGTGGAGTTCAATTA	55.5

[a] PCR fragments correspond to those listed in supplementary Table S5.

[b] Nucleotide positions obtained from mitogenome #20, *H. r. rustica* reference sequence (HrrRS, MZ905359).

Supplementary Table 7. Oligonucleotides employed for long-range PCR amplification of barn swallow mitogenomes. The three primer pairs used to amplify the entire mitogenome in three overlapping PCR fragments.

PCR fragment	Primer name	5'np [a]	3'np [a]	Sequence 5'→ 3'	T_m (ºC)
A	Hr_118_FOR	49	68	GCAAGTATCCGCATTCCAGT	60.1
	Hr_6624 REV	6535	6554	AGGGGGAATCAGTGTGTGAA	60.4
B	Hr_6215 FOR	6215	6234	TCGTATGAGCCCACCACATA	60.4
	Hr_12655 REV	12565	12584	GCGACAACTATTGTGCTGGA	59.9
C	Hr_12374 FOR	12304	12323	CGAGCAGAAGCCAATACCTC	60.0
	Hr_694 REV	606	625	CTGTTCTGGCAAGGAATGGT	60.0

[a] Nucleotide positions obtained from mitogenome #20, *H. r. rustica* reference sequence (HrrRS, MZ905359).

SUPPLEMENTARY REFERENCES

Carter JK, Innes P, Goebl AM, Johnson B, Gebert M, Attia Z, Gabani Z, Li R, Melie T, Dart C, et al. 2020. Complete mitochondrial genomes provide current refined phylogenomic hypotheses for relationships among ten *Hirundo* species. Mitochondrial DNA B Resour. 5(3):2881-2885.

Cerasale DJ, Dor R, Winkler DW, Lovette IJ. 2012. Phylogeny of the *Tachycineta* genus of New World swallows: insights from complete mitochondrial genomes. Mol Phylogenet Evol. 63(1):64-71.

Eberhard JR, Wright TF, Bermingham E. 2001. Duplication and concerted evolution of the mitochondrial control region in the parrot genus *Amazona*. Mol Biol Evol. 18(7):1330-1342.

Grant JR, Stothard P. 2008. The CGView Server: a comparative genomics tool for circular genomes. Nucleic Acids Res. 36 (Web Server issue):W181-4. Available from: http://www.cgview.ca.

Liu S, Chen Y, Liu JD, Wu YH, Xie JH, Shen YW. 2016. Complete mitochondrial genome of Red-rumped Swallow, *Cecropis daurica* (Passeriformes: Hirundinidae). Mitochondrial DNA A DNA Mapp Seq Anal. 27(1):516-517.

Pereira L, Freitas F, Fernandes V, Pereira JB, Costa MD, Costa S, Máximo V, Macaulay V, Rocha R, Samuels DC. 2009. The diversity present in 5140 human mitochondrial genomes. Am J Hum Genet. 84(5):628-640.

Pereira SL, Grau ET, Wajntal A. 2004. Molecular architecture and rates of DNA substitutions of the mitochondrial control region of cracid birds. Genome. 47(3):535-545.

Rozas J, Ferrer-Mata A, Sánchez-DelBarrio JC, Guirao-Rico S, Librado P, Ramos-Onsins SE, Sánchez-Gracia A. 2017. DnaSP 6: DNA sequence polymorphism analysis of large data sets. Mol Biol Evol. 34(12):3299-3302.

Saunders MA, Edwards SV. 2000. Dynamics and phylogenetic implications of mtDNA control region sequences in New World jays (Aves: Corvidae). J Mol Evol. 51(2):97-109.

Urantówka AD, Kroczak A, Mackiewicz P. 2020. New view on the organization and evolution of Palaeognathae mitogenomes poses the question on the ancestral gene rearrangement in Aves. BMC Genomics. 21(1):874.

Turner A. 2006. The Barn Swallow. London, T. & A.D. Poyser.
